# Polymorphic Superparaelectric Engineering Boosting Energy Storage Capacity in BaTiO_3_‐Based Ceramics

**DOI:** 10.1002/advs.202524252

**Published:** 2026-01-22

**Authors:** Pan Liu, Xiang Ren, Jin Qian, Haihua Huang, Peng Li, Peng Fu, Jigong Hao, Huarong Zeng, Wei Li, Zhenxiang Cheng

**Affiliations:** ^1^ Laboratory of Sensitive Materials and Devices Shandong Department of Education School of Materials Science and Engineering Liaocheng University Liaocheng China; ^2^ Functional Materials Research Laboratory School of Materials Science and Engineering Tongji University Shanghai China; ^3^ State Key Laboratory of High Performance Ceramics Shanghai Institute of Ceramics Chinese Academy of Sciences Shanghai China; ^4^ Institute for Superconducting and Electronic Materials, Faculty of Engineering and Information Sciences Innovation Campus University of Wollongong North Wollongong New South Wales Australia

**Keywords:** barium titanate, dielectric ceramics, energy storage, ferroelectrics, superparaelectrics

## Abstract

Electrostatic energy storage using dielectrics plays a vital role in advanced electronics and high‐power electrical systems. While superparaelectric materials offer great potential for achieving high recoverable energy density (*W*_rec_) and efficiency (*η*), their practical applications have been hindered by intrinsically low polarization. Herein, a polymorphic superparaelectric engineering approach that simultaneously enhances polarization and breakdown strength was introduced. By constructing coexisting cubic‐orthorhombic‐tetragonal (C‐O‐T) superparaelectric states in BaTiO_3_‐based ceramics, the energy barrier for polarization switching is effectively reduced, leading to improved macroscopic polarization and reinforced breakdown endurance. As a result, the optimized polymorphic superparaelectric ceramics achieve a high *W*_rec_ of 9.8 J cm^−3^ and *η* of 88.5% under 820 kV cm^−1^, along with exceptional stability‐frequency stability with *W*_rec_ variation within ±0.6% and *η* variation within ±3.3% from 1 to 400 Hz, and fatigue stability with both *W*_rec_ and *η* varying below ±0.3% over 10^5^ cycles. These results underscore the material's promise for high‐energy pulsed power applications and establish a new design strategy for next‐generation dielectric capacitors.

## Graphical abstract

Herein, Ca^2+^ incorporation promotes the coexistence of CaTiO_3_‐/BaTiO_3_‐derived paraferroelectric states, stabilizing cubic‐orthorhombic‐tetragonal polymorphic superparaelectric phases. This minimizes polarization energy barriers, facilitating full polarization saturation without compromising efficiency. Furthermore, Ca^2+^ doping induces lattice distortion and suppresses A‐site vacancy migration via ionic size effects and bandgap modulation. These synergistic effects enable the significant enhancement of maximum polarization and breakdown strength.

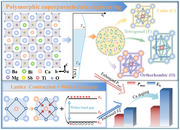

## 1 Introduction

As global energy consumption continues to rise, the integration of renewable energy sources is driving the need for advanced energy storage technologies [1, 2, 3]. Among these, dielectric capacitors have gained attention for their ability to enable miniaturized, integrated, and high‐power pulsed energy storage systems [4]. These ceramic‐based devices store energy electrostatically, supporting ultra‐fast charging and discharging rates [5, 6]. BaTiO_3_ (BT), a well‐known ferroelectric (FE) ceramic with a perovskite structure, has been widely studied owing to its high dielectric constant (*ε*_r_), low dielectric loss (tan*δ*), and excellent thermal stability [7, 8]. However, despite these merits, its use in pulsed power devices remains constrained by significant hysteresis during charging‐discharging cycles and the inherent trade‐off between maximum polarization (*P*_ma_ *_x_*) and breakdown strength (*E*_b_), which collectively limit energy storage performance [9, 10].

In recent years, superparaelectrics (SPEs)–transient relaxor states occurring between the dielectric maximum temperature (*T*_m_) and the Burns temperature (*T*_B_)‐have emerged as attractive candidates for energy storage [11, 12, 13]. These systems exhibit strong capacitive responses originating from dynamic nanopolar clusters, whose energy barriers are on the order of thermal energy *kT* [14, 15, 16, 17]. This feature allows rapid polarization switching, contributing to high recoverable energy density (*W*_rec_) and efficiency (*η*) [18, 19]. Moreover, the controlled weakening of inter‐cluster coupling in the SPE regime helps suppress losses, further improving energy storage across multiple material systems [20, 21, 22]. Still, the relatively low *P*_ma_ *_x_* of relaxor ferroelectrics (RFEs) fundamentally restricts *W*_rec_, suggesting that single‐phase SPE configurations are inadequate for optimal performance.

**FIGURE 1:**
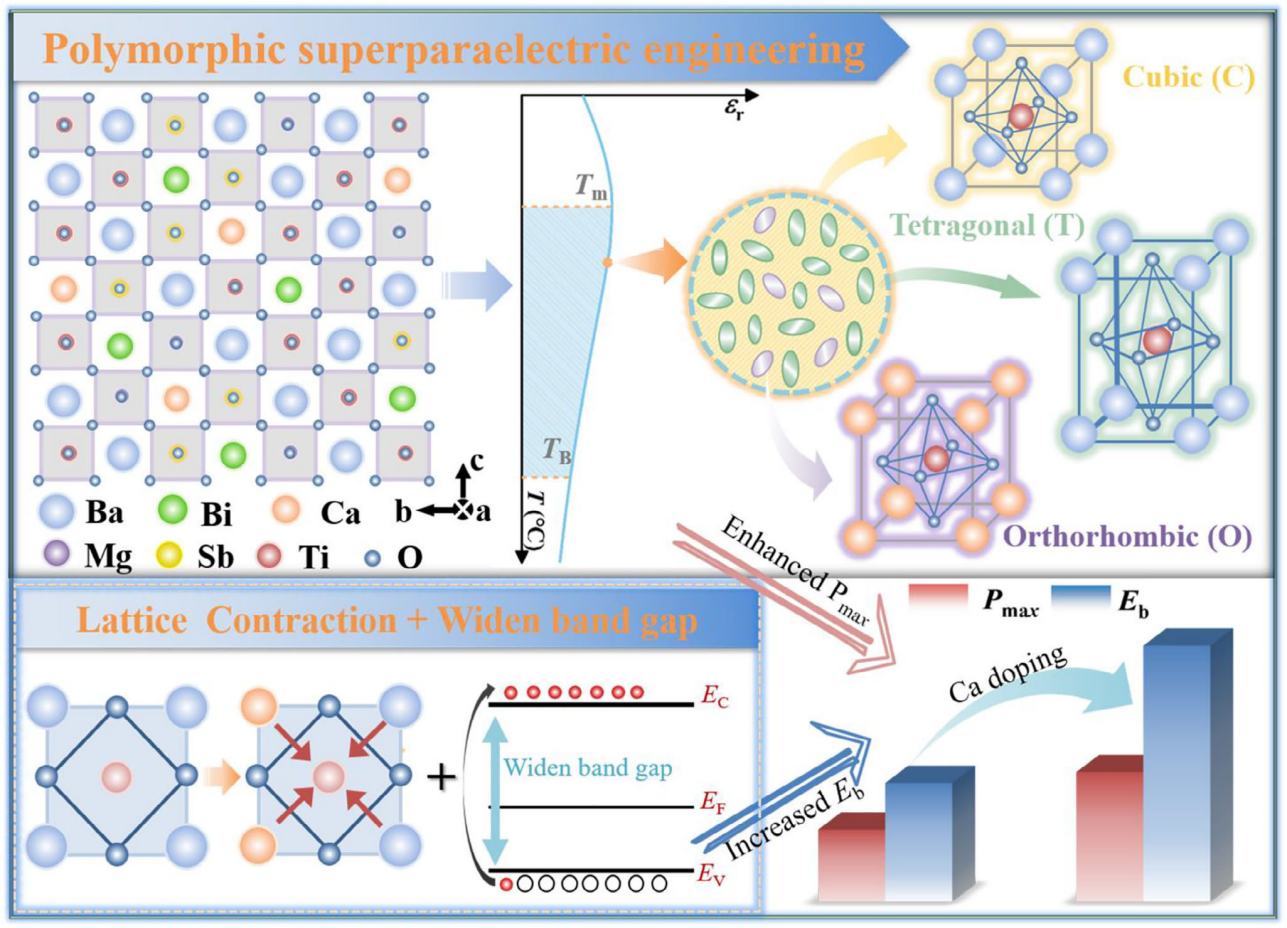
Schematic diagram of the polymorphic superparaelectric engineering strategy for simultaneously enhancing *P*_ma_ *_x_* and *E*_b_ in dielectric energy storage ceramics.

Recent advances in lead‐free dielectric films have shown that locally coexisting multiphase SPE structures can effectively lower polarization reversal barriers, thereby synergistically boosting both *P*_ma_ *_x_* and *E*_b_ while maintaining low hysteresis [23]. Inspired by this, we propose a new material design strategy termed polymorphic superparaelectric engineering. We demonstrate this approach in a series of 0.92Ba_1−_ *_x_* Ca*_x_* TiO_3_‐0.08Bi(Mg_2/3_Sb_1/3_)O_3_ (B_1−_ *_x_* C*_x_* T‐BMS) ceramics, where Ca^2+^ incorporation promotes the coexistence of paraelectric states derived from CaTiO_3_ and BaTiO_3_. This results in the stabilization of polymorphic superparaelectric phases (cubic‐orthorhombic‐tetragonal, C‐O‐T), which minimizes polarization energy barriers and facilitates full polarization saturation without compromising *η*. Additionally, Ca^2+^ doping introduces lattice distortion and suppresses A‐site vacancy migration through ionic size effects and bandgap (*E*_g_) modulation, collectively enhancing breakdown resistance [24, 25, 26]. As summarized in Figure 1, this strategy enables simultaneous enhancement of *P*_ma_ *_x_* and *E*_b_, leading to a remarkable *W*_rec_ of 9.8 J cm^−3^ and *η* of 88.5% at 820 kV cm^−1^. These results underscore polymorphic phase design as a viable and effective pathway toward high‐performance energy storage ceramics.

## 2 Results and Discussion

### 2.1 Polymorphic Superparaelectric Phase Structure Coexisting with High *P*_ma_ *_x_*

XRD patterns of all compositions confirm pure perovskite structures without secondary phases (Figure S1), indicating successful incorporation of Ca^2+^ into the BT lattice [27, 28]. A systematic shift of the (200) diffraction peak toward higher angles reflects lattice contraction, consistent with the substitution of smaller Ca^2+^ (1.34 Å) for larger Ba^2+^ (1.61 Å) ions. To quantitatively determine the phase evolution, Rietveld refinement was performed on representative samples (*x* = 0 and 0.18), as shown in Figure S2. The refinement results, with satisfactory reliability factors, clearly reveal a transition from C‐T to C‐O‐T phase coexistence with increasing Ca content. Specifically, the C‐ and T‐phase fractions accounted for 49.7% and 50.3% at *x* = 0, while the C‐phase fraction increased to 54.6%, the T‐phase fraction decreased to 35%, and an O‐phase emerged with a 10.4% fraction at *x* = 0.18. This emergence of polymorphic superparaelectric states effectively reduces the energy barrier for reversal polarization. Notably, the sample with *x* = 0.18 exhibits a higher fraction of the C‐phase, which disrupts the long‐range ferroelectric ordering. This structural change, along with the stabilization of orthorhombic and tetragonal phases, results in polymorphic superparaelectric states. Consequently, the energy barrier for polarization reversal is effectively reduced, as a result, the polarization switching is enhanced under high electric fields [29].

Raman spectroscopy was employed to probe the local structural evolution across the series (Figure S3) [28]. All compositions exhibit broad and diffuse Raman bands, characteristic of A‐site cation disorder caused by the random occupation of Ba^2+^, Bi^3+^, and Ca^2+^ [24]. With increasing Ca content, the A‐site vibration mode near 180 cm^−1^ becomes progressively broader, indicating enhanced cationic disorder [30]. The intensity of the peak at 306 cm^−^^1^, assigned to the B_1_+E (LO+TO) “silent” mode and indicative of tetragonal phase, gradually weakens, suggesting increased structural symmetry [31]. Furthermore, the A_1_(TO_3_)/E(TO) mode shifts from 514 cm^−1^ (*x* = 0) to 520 cm^−1^ (*x* = 0.18), implying strengthened bonding between A‐site cations and oxygen anions, in agreement with XRD analysis [32].

Grain size and microstructure play crucial roles in determining the phase structure and functional properties of BT‐based ceramics [33, 34]. SEM images (Figures S4 and S5) show well‐developed grains with clear boundaries and low porosity, providing a robust microstructure conducive to high breakdown strength. Dielectric properties were characterized from −50°C to 150°C (Figure S6). All compositions exhibit frequency dispersion and diffuse phase transition behavior, typical of relaxors (Figure S6a–g). The maximum dielectric constant (*ε*_m_) initially increases and then decreases with doping, a trend potentially influenced by grain size variation [35]. The composition with *x* = 0.18 shows the highest *ε*_m_, implying a stronger polarization response under applied electric fields [22]. The corresponding *T*_m_ also shifts, possibly due to the incorporation of CaTiO_3_, which possesses a higher intrinsic *T*_m_ (Figure S6h). The temperature range between *T*_m_ and *T*_B_ defines the SPE region, where highly dynamic polar nanoregions (PNRs) coexist with a nonpolar matrix [15, 22]. As derived from Figure S7, all samples reside in the SPE state at room temperature. Compared to the undoped ceramic, the solid solution with *x* = 0.18 introduces an orthorhombic SPE phase into the existing T‐C SPE matrix, effectively realizing the polymorphic superparaelectric structure, which is further corroborated by the XRD results.

### 2.2 Domain Structure with Polymorphic Superparaelectric Phases, Reducing Polarization Reversal Energy Barrier

**FIGURE 2:**
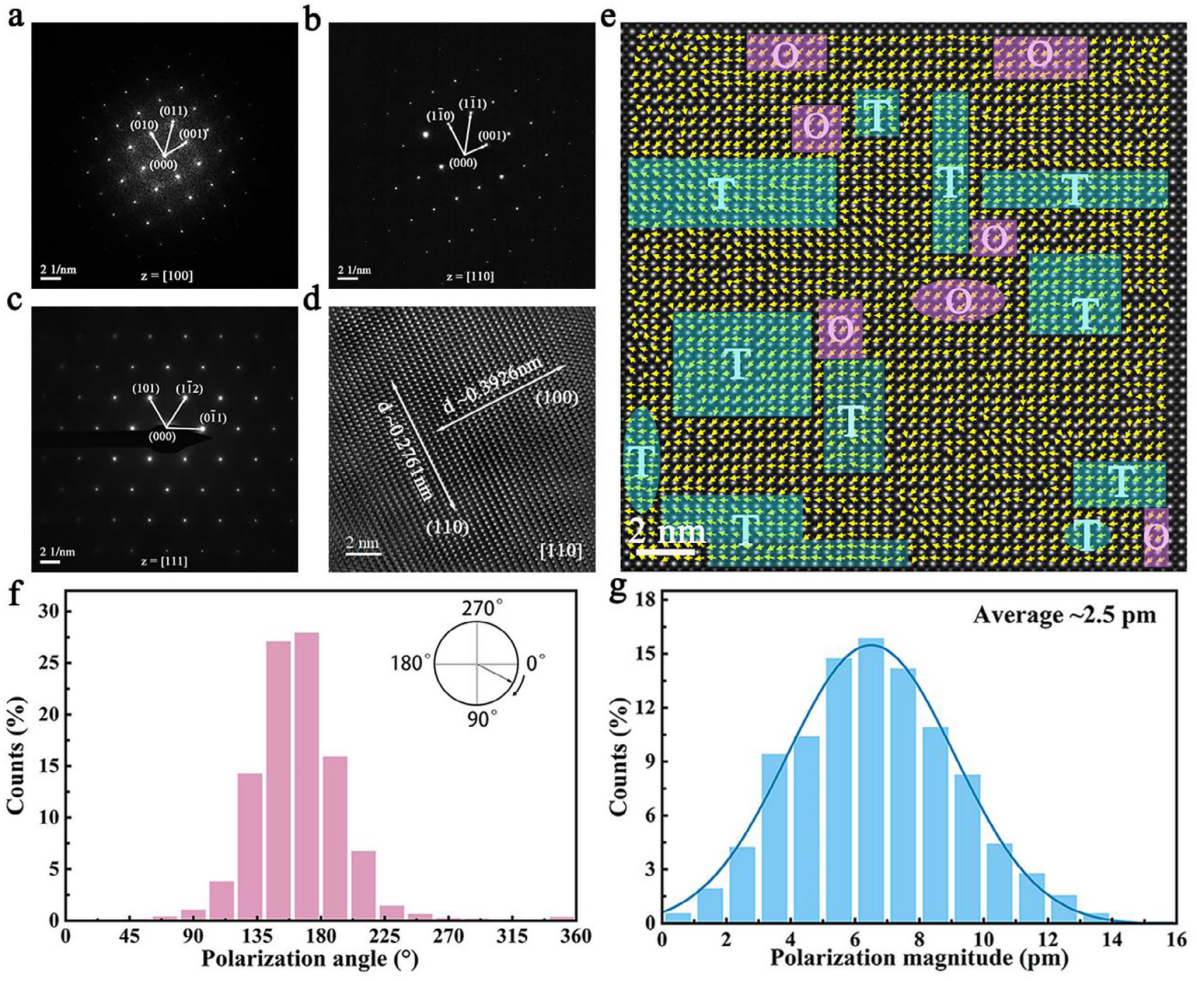
Domain structure and polarization characteristics of the *x* = 0.18 ceramic. (a–c) SEAD images along the [100], [110], and [111] zone‐axes; (d) HR‐TEM image viewed along the [110] direction; (e) Atomic displacement vector mappings derived from HAADF STEM images along the [100] direction; (f,g) Statistical distributions of (f) polarization angle and (g) polarization magnitude along the [100] direction.

Domain structure and polarization configuration play a crucial role in enhancing the breakdown strength (*E*_b_) and delaying polarization saturation [36]. To probe these features in the optimized composition, selected‐area electron diffraction (SAED) and high‐resolution transmission electron microscopy (HR‐TEM) were carried out on the *x* = 0.18 ceramic. SAED patterns collected along the [100]_c_, [110]_c_, and [111]_c_ zone axes (Figure 2a–c) show no superlattice reflections, indicating that the local polarization is primarily governed by cation displacements rather than long‐range periodic ordering [37]. HR‐TEM imaging along the [110]_c_ direction (Figure 2d) reveals well‐defined lattice fringes with interplanar spacings of approximately 0.3926 nm for (001) and 0.2761 nm for (110). The ratio d_(001)_/d_(110)_ ≈ 1.422 exceeds the ideal cubic value (√2 ≈ 1.414), confirming a pseudocubic structure with local tetragonal distortion [38]. Such local lattice distortions, commonly induced by atomic size, mass, and electronegativity mismatch in multi‐cation systems, contribute positively to polarization enhancement [39, 40, 41].

Further analysis via inverse fast Fourier transform (IFFT) on regions marked in Figure S8a reveals significant lattice strain (Figure S8b,c). These distortions help suppress dipole moment reduction, thereby supporting a higher *P*_ma_ *_x_* [42]. To directly resolve the local polarization arrangement enabled by polymorphic superparaelectric engineering, atomic‐resolution high‐angle annular dark‐field (HAADF) STEM was performed along the [100] direction. Yellow arrows in the images represent polarization vectors pointing from the B‐site cation center to the A‐site cation, illustrating both the direction and magnitude of cation off‐centering [43]. By correlating the projected polarization vector directions with known phase symmetry attributes, vectors aligned with [001]_c_ are assigned to the tetragonal (T) phase, whereas those oriented along [011]_c_ correspond to rhombohedral/orthorhombic (R/O) phases [44, 45, 46]. A schematic unit cell illustration along the [100] projection (Figure S8d) clarifies this assignment. The HAADF‐STEM image in Figure 2e, analyzed using 2D Gaussian peak fitting, confirms the coexistence of C‐, O‐, and T‐phase nanodomains. Here, the C‐phase acts as the matrix, while O‐ and T‐type nanodomains percolate throughout, collectively facilitating polarization switching [23, 47]. A close‐up view of the region highlighted in Figure S8e, presented in Figure S8f, captures a transition state between differently oriented nanodomains during polarization reversal. Such transitional configurations significantly lower the energy barrier for domain switching, directly contributing to enhanced energy storage performance [5, 48].

The polarization angle distribution mapped in Figure 2f exhibits a broad spread, attributable to variations in ionic radii and ferroelectric activity among A‐ and B‐site cations, as further corroborated by Figure S9a. In addition, the polarization magnitude distribution (Figure 2g) shows an average value of approximately 2.5 pm calculated from Figure S9b, following a near‐normal distribution. The presence of near‐zero polarization amplitudes supports the argument that the C‐phase matrix restrains polarization rotation, mitigates internal stress, and thus delays polarization saturation [44, 49]. Together with earlier Raman analysis, these results underscore the role of A‐site (Ba/Bi/Ca) cation heterogeneity in promoting local structural disorder. In relaxor ferroelectrics in the SPE state, interdomain coupling weakens such that the energy barrier for polarization switching becomes comparable to or lower than the thermal energy *kT* [1, 17, 22, 50]. This permits highly dynamic reorientation of ultrafine nanodomains, yielding higher *η* compared to conventional relaxor ferroelectrics [18, 19]. The present work demonstrates that polymorphic superparaelectric engineering further reduces the polarization reversal barrier, opening new avenues for tailoring material properties in high‐performance energy storage applications.

### 2.3 Energy Storage and Pulse Performance Optimized Through Polymorphic Superparaelectric Engineering

**FIGURE 3:**
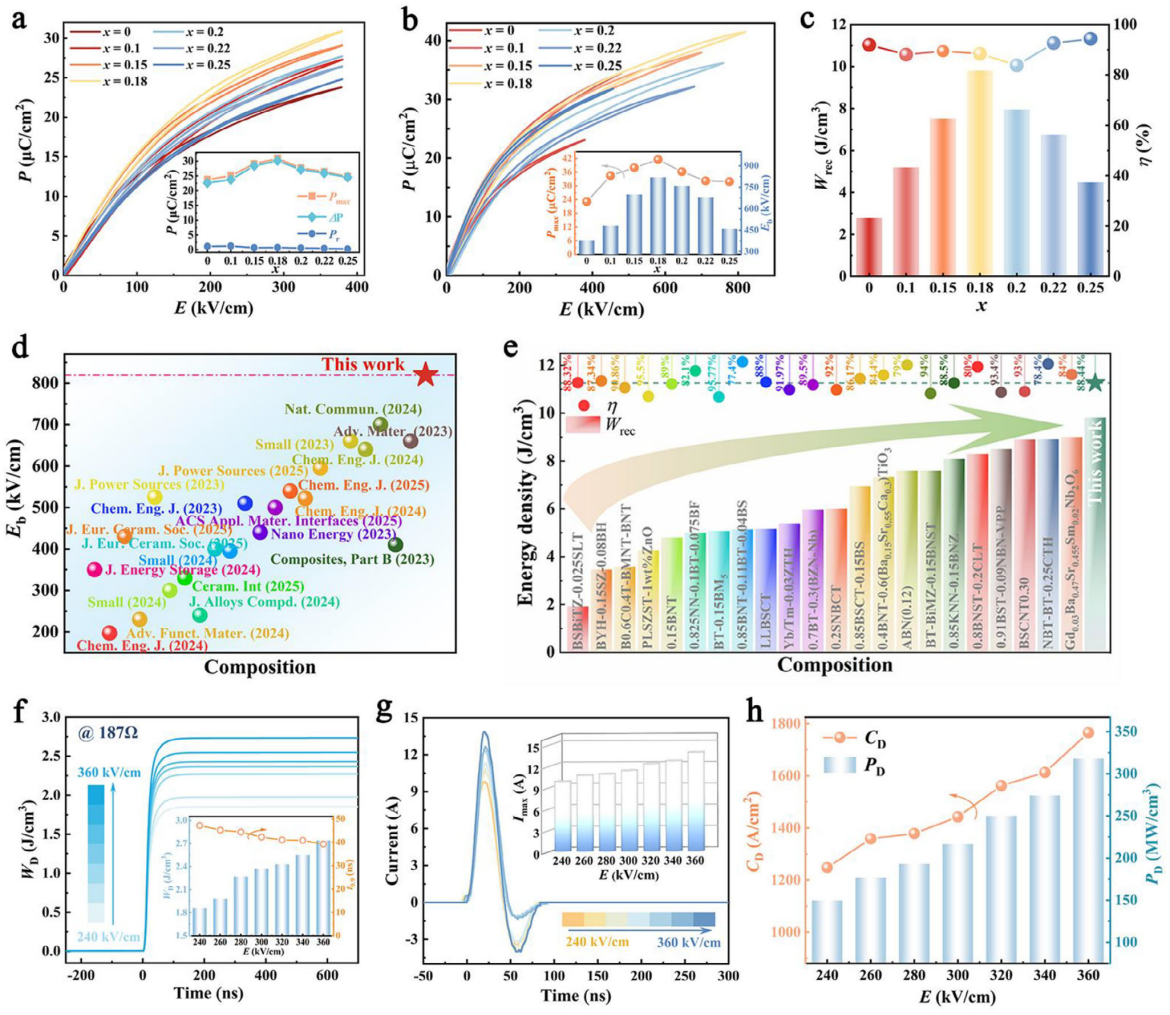
Energy storage and charge–discharge performance of B_1‐_ *_x_* C*_x_* T‐BMS ceramics. (a) Unipolar *P*‐*E* loops measured at 380 kV cm^−1^, with inset showing composition dependence of *P*_ma_ *_x_*, Δ*P*, and *P*_r_; (b) Unipolar *P*‐*E* loops measured at respective *E*_b_, with inset showing variations of *P*_ma_ *_x_* and *E*_b_ with composition *x*; (c) *W*_rec_ and *η* as a function of *x* at *E*_b_; (d,e) Comparision of (d) *E*_b_ and (e) *W*_rec_ and *η* between the *x* = 0.18 ceramic and other recently reported lead‐free systems; (f) Overdamped *W*_D_‐*t* curve for B_0.82_C_0.18_T‐BMS ceramics; inset shows electric‐field‐dependent current response); (g) Underdamped discharge current curve, inset illustrates current variation with electric field; (h) Electric field dependence of *C*_D_ and *P*_D_.

To evaluate the energy storage performance of the synthesized ceramics, unipolar *P*‐*E* hysteresis measurements were conducted on all compositions. Figure 3a displays the slim *P*‐*E* loops measured at 380 kV cm^−1^, with the inset summarizing the composition dependence of *P*_ma_ *_x_*, *P*_r_, and Δ*P* ( = *P*_ma_ *_x_* – *P*_r_). Both *P*_ma_ *_x_* and *P*_r_ initially increase with *x*, reaching a maximum at *x* = 0.18, before decreasing at higher Ca concentrations. The *x* = 0.18 ceramic achieves a high *P*_ma_ *_x_* of 30.8 µC cm^−2^, which is about 1.3 times that of the undoped sample (23.8 µC cm^−2^), underscoring the efficacy of polymorphic superparaelectric engineering in enhancing polarization. This improvement in *P*_ma_ *_x_* stems from two competing effects arising from the changing phase composition. On one hand, the introduction of polymorphic superparaelectric states lowers the energy barrier for domain switching, and the weakened correlation among PNRs promotes a more complete electric field response. On the other hand, the overall dipole moment tends to decrease with heavy Ca doping. The former effect dominates in the range 0≤ *x* ≤0.18, whereas the latter becomes more influential beyond this composition.

Figure 3b presents the unipolar *P*‐*E* loops measured at *E*_b_, with the inset depicting the variation of *E*_b_ and *P*_ma_ *_x_* with *x*. The polymorphic nanodomain configuration facilitates a considerable increase in dielectric permittivity *ε*_r_ while maintaining low tan*δ*, attributable to enhanced domain wall mobility and polarization rotation [51, 52]. As shown in Figure S10, *P*_ma_ *_x_* increases monotonically with the electric field, while *P*_r_ remains low, leading to a large *Δ*P. Ultimately, the *x* = 0.18 ceramic exhibits a synergistic improvement in *E*_b_ (820 kV cm^−1^) and *P*_ma_ *_x_* (41.5 µC cm^−2^), yielding an outstanding *W*_rec_ of 9.8 J cm^−3^ and a high *η* of 88.5% (Figure 3b, inset, and Figure 3c). Notably, even under such a high electric field, the *η* is only 3.9% lower than that of the undoped ceramic, making it suitable for a broad range of applications. Energy dissipation in the B_1‐_ *_x_* C*_x_* T‐BMS system is primarily attributed to the combined effects of rapid PNR switching and leakage currents [53, 54].

A comparison of the performance parameters (*E*_b_, *W*_rec_, and *η*) with other recently reported ceramic systems (Figure 3d,e; Table S1) confirms that the present material ranks among the best‐performing energy storage ceramics, highlighting its promising application potential. The *x* = 0.18 ceramic was further subjected to pulse discharge characterization. Key metrics include the discharge energy density (*W*_D_), current density (*C*_D_), power density (*P*_D_), and the discharge time *t*_0.9_ (time to release 90% of the stored energy), defined as:

(1) $$W_{D}=\frac{R\int I^{2}\left(t\right)\mathrm{dt}}{V}$$

(2) $$C_{D}=\frac{I_{\mathrm{ma}x}}{S}\;$$

(3) $$\;P_{D}=\frac{EI_{\mathrm{ma}x}}{2S}\;$$

where *R* is the load resistance (187 Ω), *V* is the sample volume, and *S* is the electrode area [55, 56].

The overdamped discharge current profile and the corresponding *W*_D_ are shown in Figure S11 and Figure 3f, respectively. The inset in Figure 3f illustrates the electric‐field dependence of *W*_D_ and *t*_0.9_ under overdamped conditions. The polymorphic superparaelectric design introduces a high density of highly responsive PNRs, leading to an ultrafast discharge time *t*_0.9_ of ∼39.2 ns. *W*_D_ increases steadily with the applied field, reaching a maximum of 2.74 J cm^−3^ at 360 kV cm^−1^. Figure 3g displays the underdamped discharge current waveform at room temperature, together with the corresponding *I*_ma_ *_x_* (inset), while Figure 3h plots the derived *C*_D_ and *P*_D_. At 360 kV cm^−1^, the ceramic achieves a remarkable *C*_D_ of 1765.98 A cm^−2^ and a *P*_D_ of 317.88 MW cm^−3^. These outstanding pulse charge–discharge characteristics underscore the material's strong potential for use in high‐power pulsed capacitors.

### 2.4 Origin of the Enhanced *E*_b_

**FIGURE 4:**
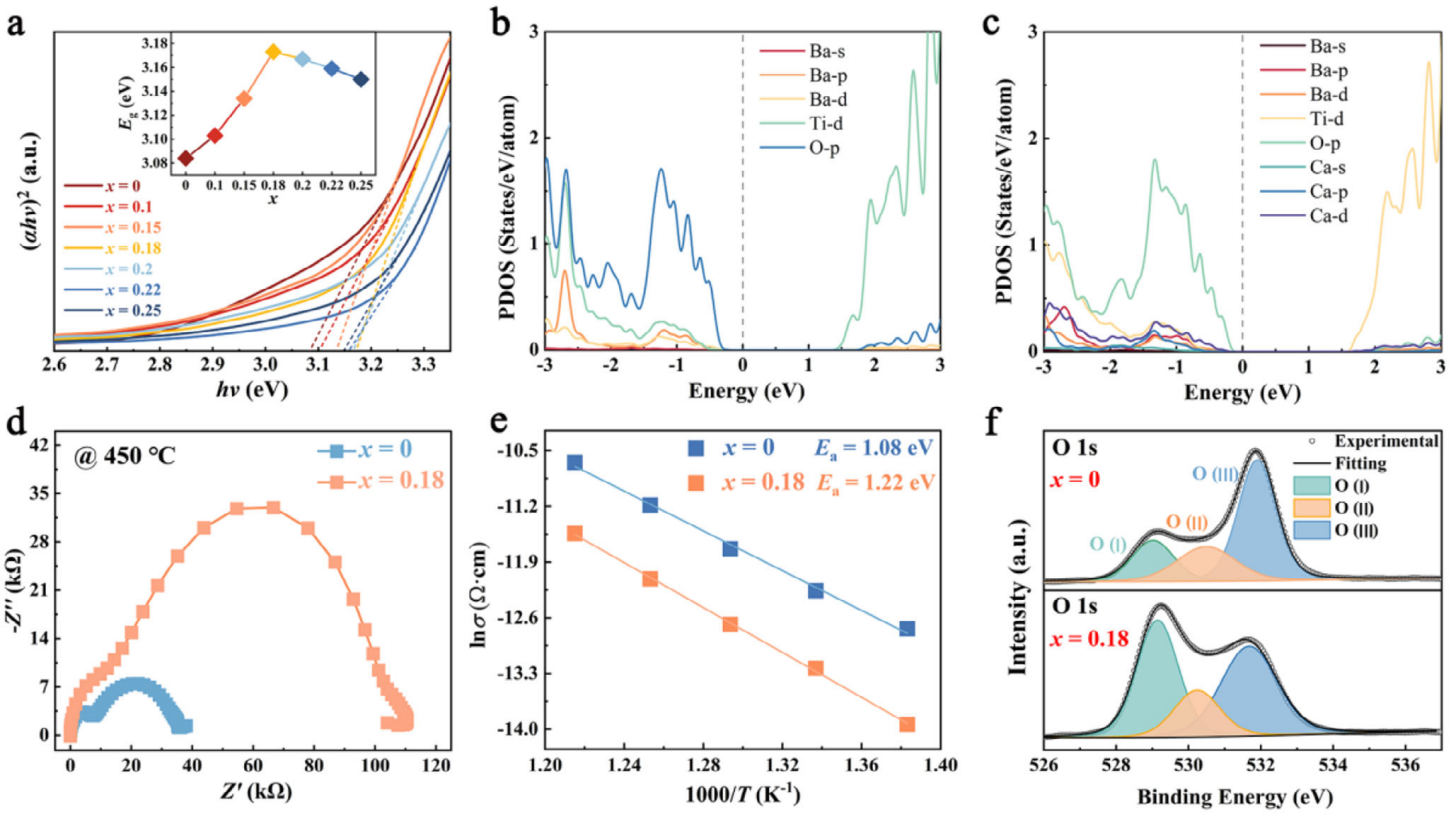
Electronic structure and electrical characterization of B_1‐_ *_x_* C*_x_* T‐BMS ceramics. (a) Tauc plots of (*αhν*)^2^ vs. *hν*, inset is the corresponding *E*_g_ values; (b,c) Density of states for (b) BT and (c) BT‐Ca (the dashed line denotes the Fermi level); (d) Impedance spectra of *x* = 0 and *x* = 0.18 ceramics at 450°C; (e) Arrhenius plots of ln𝜎 vs. 1000/*T* for *x* = 0 and *x* = 0.18 compositions; (f) O 1s XPS spectra of undoped and Ca‐doped ceramics.

The high *E*_b_ exhibited by these ceramics stems from a combination of structural and electronic factors, including lattice distortion, uniform grain morphology, well‐defined grain boundaries, a wide *E*_g_, and low tan*δ* [57]. To investigate the origin of this enhancement, UV–vis absorption spectra were recorded for all compositions, as shown in Figure S12 and Figure 4a. The optical bandgap was determined using the Tauc relation:

(4) $${\left(\alpha h\nu \right)}^{2}=A\left(h\nu -E_{g}\right)$$

where *hν*, *α*, and *A* denote the incident photon energy, absorption coefficient, and a proportionality constant, respectively [58]. The *E*_g_ values obtained from linear fitting are summarized in the inset of Figure 4a. The *E*_g_ initially increases with Ca content, reaching a maximum at *x *= 0.18, and then decreases at higher doping levels. This enhancement is attributed to the substitution of Ba^2+^ by smaller Ca^2+^ ions, which induces lattice contraction and strengthens chemical bonding, thereby widening the gap between valence and conduction bands and improving the intrinsic breakdown strength [59]. Elemental mapping via EDS (Figure S13) further confirms the homogeneous distribution of Ca in the *x *= 0.18 ceramic, indicating its successful incorporation into the lattice. Such chemical uniformity helps mitigate local field concentrations, contributing to the improved *E*_b_ [49].

To probe the effect of Ca doping on electronic structure, density functional theory (DFT) calculations were performed. The base structure of BaTiO_3_ (Figure S14a) contains two Ba, two Ti, and six O atoms per unit cell. A 2 × 2 × 2 supercell with the composition Ca_3_Ba_13_Ti_16_O_48_ was constructed to model the Ca‐doped system, where select Ba sites are replaced by Ca (Figure S14b). After structural optimization, the partial density of states (PDOS) was analyzed to understand the orbital contributions to the band structure (Figure 4b,c). Both pristine and Ca‐doped BaTiO_3_ exhibit semiconductor behavior, with calculated bandgaps of 1.65 and 1.76 eV, respectively (Figure S14c,d). The valence band dispersion is similar for both systems, dominated by O 2p states. However, within a limited energy range, the conduction band of the doped system exhibits a higher electron density of states, suggesting an increase in the electron average effective mass (m^*^), estimated as m^*^(0.18) = 0.52 for the doped system compared to m^*^(0) = 0.35 for the undoped case. Since electronic conductivity is inversely related to the carrier effective mass, the doped ceramic exhibits reduced electrical conduction.

It is well established that *E*_b_ correlates positively with the total resistance (*R*_b_) of the ceramic, which includes contributions from grain (*R*_g_) and grain boundary (*R*_gb_) regions [58, 60]. Impedance spectroscopy was conducted as a function of temperature for *x *= 0 and *x *= 0.18 samples (Figure S15). Both compositions exhibit two semicircular arcs, indicating similar electrical heterogeneity dominated by grain and grain boundary effects. The arc diameters decrease with increasing temperature, reflecting a reduction in overall resistivity. At 450°C, the *x *= 0.18 ceramic exhibits a larger *R*_b_ than its undoped counterpart (Figure 4d). The activation energy (*E*_a_) of conductivity, which reflects the ease of carrier migration, was calculated via the Arrhenius law:

(5) $$\sigma =\sigma_{0}\exp\left(-\frac{E_{a}}{k_{B}T}\right)$$

where *k*_B_, *T*, *σ*_0_, and *σ* represent the Boltzmann constant, absolute temperature, pre‐exponential factor, and reciprocal of *R*_b_, respectively [40]. According to the fitting results (Figure 4d), the sample *x* = 0.18 exhibits a higher *E*_a_ of 1.22 eV (Figure 4e). This increased activation energy effectively suppresses the formation and migration of oxygen vacancies, thereby enhancing the breakdown resistance of the materials. This result confirms that Ca incorporation enhances the electrical impedance of the ceramic, consistent with predictions from first‐principles calculations.

Figure 4f shows the XPS spectra of the two samples. The fitted curves consist of three main sub‐peaks: O (I) (lattice oxygen), O (II) (oxygen vacancies), and O (III) (surface‐adsorbed oxygen). The ratio of oxygen vacancies (V_O_˙˙) to lattice oxygen (O_L_) reflects the relative concentration of oxygen vacancies. The V_O_˙˙/O_L_ ratio for the *x* = 0.18 sample is 0.44, lower than that of the undoped component (1.18), confirming that Ca doping effectively reduces the oxygen vacancy content in the material. Collectively, these experimental and theoretical results demonstrate that Ca^2+^ doping enhances the breakdown performance through multiple mechanisms, offering a viable route for designing high‐performance functional ceramics.

### 2.5 Excellent Charge/Discharge Stability

**FIGURE 5:**
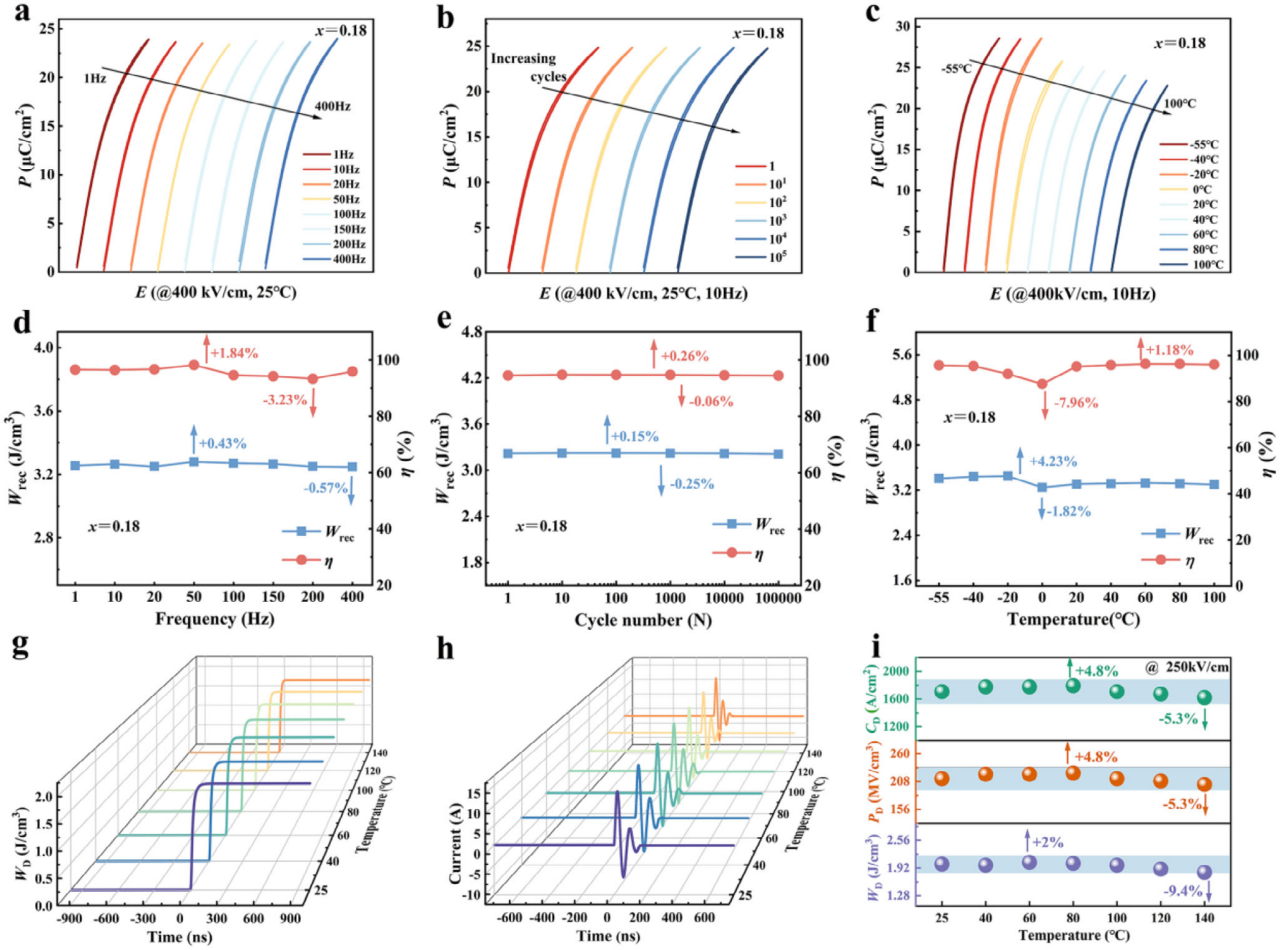
Energy storage and pulsed charge–discharge performance of the optimal composition (*x* = 0.18). (a–c) Unipolar *P*‐*E* loops under varying (a) frequency, (b) fatigue cycles, and (c) temperature; (d) Frequency dependent curves of the *W*_rec_ and *η* at 400 kV cm^−1^ and 25°C; (e) Cyclic endurance of *W*_rec_ and *η* over 10^5^ cycles at 400 kV cm^−1^, 10 Hz, and 25°C; (f) Thermal stability of *W*_rec_ and *η* at 400 kV cm^−1^ and 10 Hz; (g) Temperature‐dependent overdamped discharge energy density profiles at 250 kV cm^−1^; (h) Underdamped discharge current waveforms at different temperatures under 250 kV cm^−1^; (i) Corresponding *W*_D_, *P*_D_, and *C*_D_ as a function of temperature.

To assess performance under realistic operating scenarios, the practical pulsed characteristics of the optimal *x* = 0.18 ceramic were systematically evaluated. Figure 5a–c present the polarization behavior under varying frequency, fatigue cycling, and temperature, measured at 400 kV cm^−1^, 10 Hz, and 25°C, while Figure 5d–f summarize the corresponding variations in *W*_rec_ and *η*. The polarization response shows minimal sensitivity to frequency changes from 1 to 400 Hz, with fluctuations in *W*_rec_ and *η* limited to ±0.6% and ±3.3%, respectively. Under fatigue testing over 10^5^ cycles, both *W*_rec_ and *η* vary by less than ±0.3%, demonstrating outstanding cycling endurance. Furthermore, slim *P*‐*E* loops are maintained across a temperature range of 25°C–140°C, with *W*_rec_ and *η* varying within ±4.3% and ±8%, respectively. Such remarkable stability under varying electrical, thermal, and cyclic conditions underscores the suitability of these ceramics for practical dielectric capacitor applications.

Stable *W*_D_, *C*_D_, and *P*_D_ are equally critical for pulsed power operation. Temperature‐dependent pulsed performance was evaluated at 250 kV cm^−1^, including overdamped *I*–*t* profiles (Figure S16), overdamped *W*_D_‐*t* curves (Figure 5g), and underdamped *I‐* *t* waveforms (Figure 5h). Over the temperature range of 25°C–140°C, the values of *W*_D_, *C*_D_, and *P*_D_ exhibit only minor deviations (Figure 5i), confirming excellent thermal stability in pulsed operation – a behavior closely linked to the robust ceramic microstructure. Variable‐temperature XRD analysis (Figure S17) reveals no appreciable shift or broadening in the (200) diffraction peak, indicating high phase structural stability across the tested temperature range. Collectively, these results demonstrate that the *x* = 0.18 ceramic exhibits superior integrated performance and reliability, positioning it as a highly promising candidate for high‐energy pulsed power systems.

Based on the room‐temperature SPE state concept, we designed the B_1‐_ *_x_* C*_x_* T‐BMS system featuring polymorphic PNRs for high energy‐storage applications. Notably, the reduction in the polarization switching energy barrier enables the rapid and reversible switching of domain structures under external field induction, thereby achieving the synergistic enhancement of macroscopic polarization and breakdown endurance. This strategy can effectively break through the performance bottleneck of single‐phase SPE configurations. Building on the rich phase transition behaviors inherent to ferroelectric materials, the polymorphic SPE strategy is expected to be extended to other lead‐free ferroelectric systems.

## 3 Conclusion

In this study, we have introduced a polymorphic superparaelectric engineering strategy that enables the concurrent enhancement of *P*_ma_ *_x_* and *E*_b_ in BT‐based ceramics. By incorporating Ca^2+^ into the BT‐BMS system, we successfully stabilized coexisting C‐O‐T PNRs. The phase boundaries between these nanodomains effectively lower the energy barrier for polarization reversal, leading to a significant increase in *P*_ma_ *_x_*. The optimally doped ceramics (*x* = 0.18) deliver a high *W*_rec_ of 9.8 J cm^−3^ and *η* of 88.5% under 820 kV cm^−1^. In addition, the material exhibits excellent stability across broad temperatures (−55°C–100°C), frequency (1–400 Hz), and fatigue (10^5^ cycles) variations, together with ultrafast discharge capability (<40 ns). These results highlight the potential of B_1‐_ *_x_* C*_x_* T‐BMS ceramics as promising dielectric materials for next‐generation pulsed power capacitors designed for extreme environments. Given the general applicability of the polymorphic superparaelectric design concept, this strategy is expected to be extendable to a wide range of energy storage material systems.

## 4 Experimental Section/Methods

### 4.1 Ceramic Preparation

The solid phase synthesis method was used to prepare 0.92Ba_1‐_ *_x_* Ca*_x_* TiO_3_‐0.08Bi(Mg_2/3_Sb_1/3_)O_3_ ceramics. BaCO_3_ (Aladdin, 99.95%), CaCO_3_ (Alfa Aesar, 99.5%), TiO_2_ (Aladdin, 99.9%), Bi_2_O_3_ (Aladdin, 99.9%), MgO (Aladdin, 99.99%), and Sb_2_O_5_ (Aladdin, 99.95%) powders were used as raw materials. According to the stoichiometric ratio, the mixtures of powders were ball‐milled with ZrO_2_ balls in an anhydrous ethanol environment for 24 h. Next, the mixtures were pressed into ceramic discs with diameters of 25 mm after drying and second ball‐milled for 20 h. After the second ball milling, the dried powder was pressed into 10 mm diameter discs in which the mixture had a mass fraction of 5%–8% polyvinyl alcohol (PVA) binder. The green pellets, which were removed the PVA at 610°C, were sintered at 1250°C–1300°C for 2 h in an air atmosphere to obtain dense bulk ceramics.

### 4.2 Phase and Microstructure Characterizations

The samples were polished, and X‐ray diffraction (XRD) data were collected by an X‐ray diffractometer (XRD, SmartLab 9kW 03030502, Japan) to characterize the phase structure of the samples, which were further refined by Rietveld using the GSAS software to obtain detailed physical phase information of the samples. Local structural phase transitions or distortions of the samples were measured by laser confocal Raman microspectroscopy (Renishaw in Via, UK). Field Emission Scanning Electron Microscopy (FE‐SEM, Merlin Compact, Germany) was used to visualize the surface morphology and elemental distribution of the samples, and the grain size distribution of the samples was counted using by Nano Measurer software. Bright field imaging and selected area electron diffraction (SAED) were acquired using transmission electron microscopy (TEM, FEI Talos F200X, USA). The domain morphology and lattice stripes of the materials at the nanoscale were observed using the high‐resolution TEM (HR‐TEM) images. Atomic‐resolution high‐angle annular dark‐field (HAADF) images were acquired using an atomic‐resolution STEM (an aberration‐corrected Titan Themis G2 microscope), and the accurate polarization vectors in the STEM images were clarified via 2D Gaussian peak fitting. The angles of the polar vectors were calculated using customized MATLAB scripts. Using a UV–vis–NIR spectrophotometer (Hitachi, UH4150, Japan) measured the light absorption properties of the ceramics.

Electrical property testing: For electrical property testing, both sides of the ceramics were coated with silver paste and sintered at 610°C for 20 min. After that, the temperature dependence of *ε*_r_ and tan*δ* at different frequencies was measured in the range of −50°C to 150°C using a high and low temperature dielectric test system (HCT 1821, Tongguo Technology, China). After thinning and polishing the samples to ∼0.4 mm, complex impedance plots of the samples at different temperatures were characterized by a broadband dielectric/impedance spectrometer (concept, Novocontrol, Germany). Unipolar *P–E* curves were recorded using a ferroelectric instrument (TF Analyzer 3000, Germany). The pulse performance tests of the samples were carried out by a high‐voltage charge–discharge measuring instrument (CFD‐003 plus, Tongguo Technology, China).

## Conflicts of Interest

The authors declare no conflicts of interest.

## Supporting information




**Supporting Fil:e** advs73973‐sup‐0001‐SuppMat.docx.

## Data Availability

The data that support the findings of this study are available from the corresponding authors upon reasonable request.
