# Supplementary material for: Polymorphic Superparaelectric Engineering Boosting Energy Storage Capacity in BaTiO3‐Based Ceramics
Source: Adv Sci (Weinh). 2026 Jan 22:e24252. Online ahead of print. doi: 10.1002/advs.202524252 (PMC13325600; doi:10.1002/advs.202524252)
Supplement: Supplementary file 1 — Supporting Fil:e advs73973‐sup‐0001‐SuppMat.docx. [file ADVS-9999-e24252-s001.docx]

Supporting Information

Polymorphic Superparaelectric Engineering Boosting Energy Storage Capacity in BaTiO_3_-based Ceramics

Pan Liu, Xiang Ren, Jin Qian, Haihua Huang, Peng Li, Peng Fu, Jigong Hao,* Huarong Zeng,* Wei Li,* and Zhenxiang Cheng*


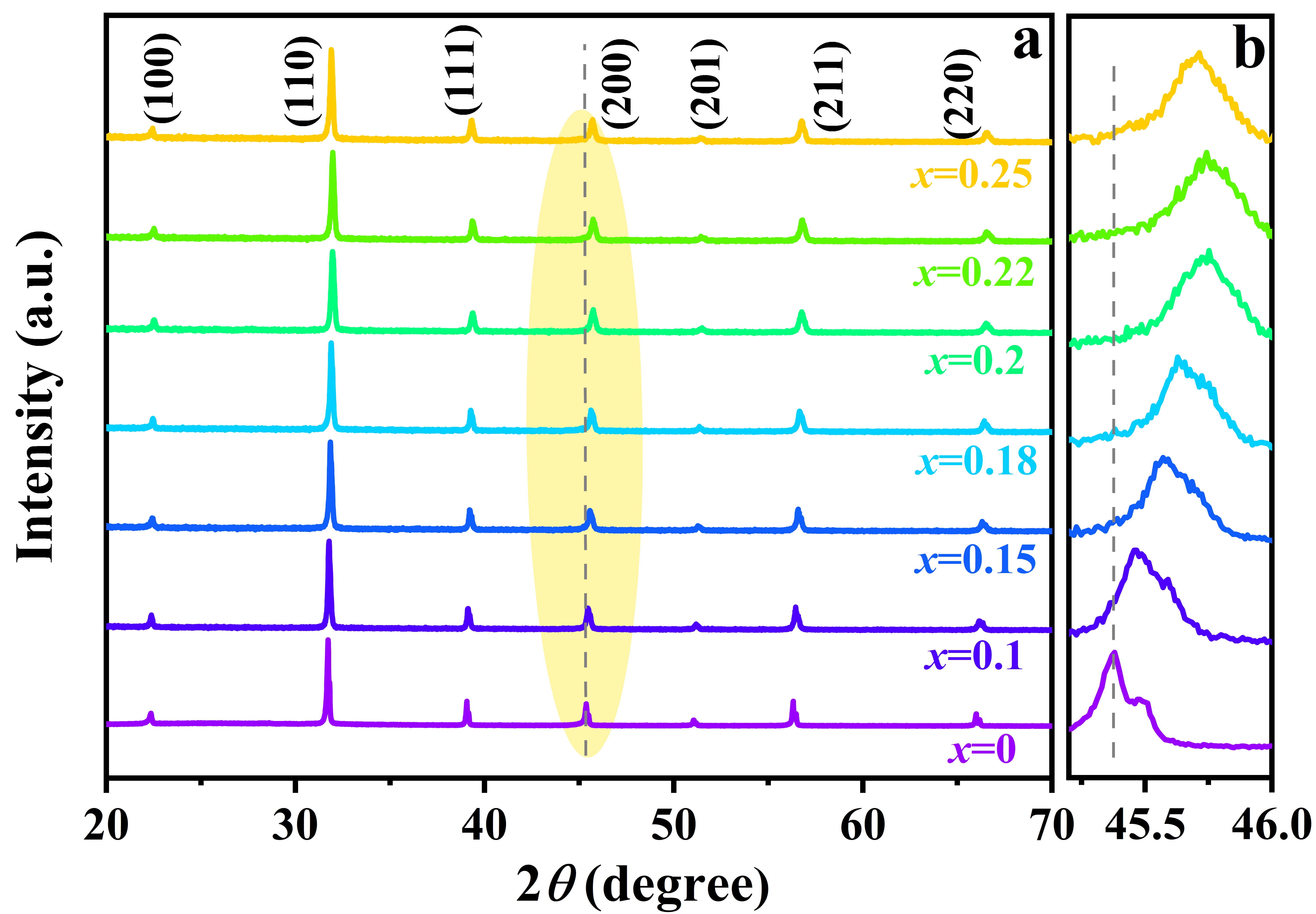


Figure S1. a) XRD patterns of B_1-_*_x_*C*_x_*T-BMS ceramics; b) enlarged image of (200) diffraction peak.


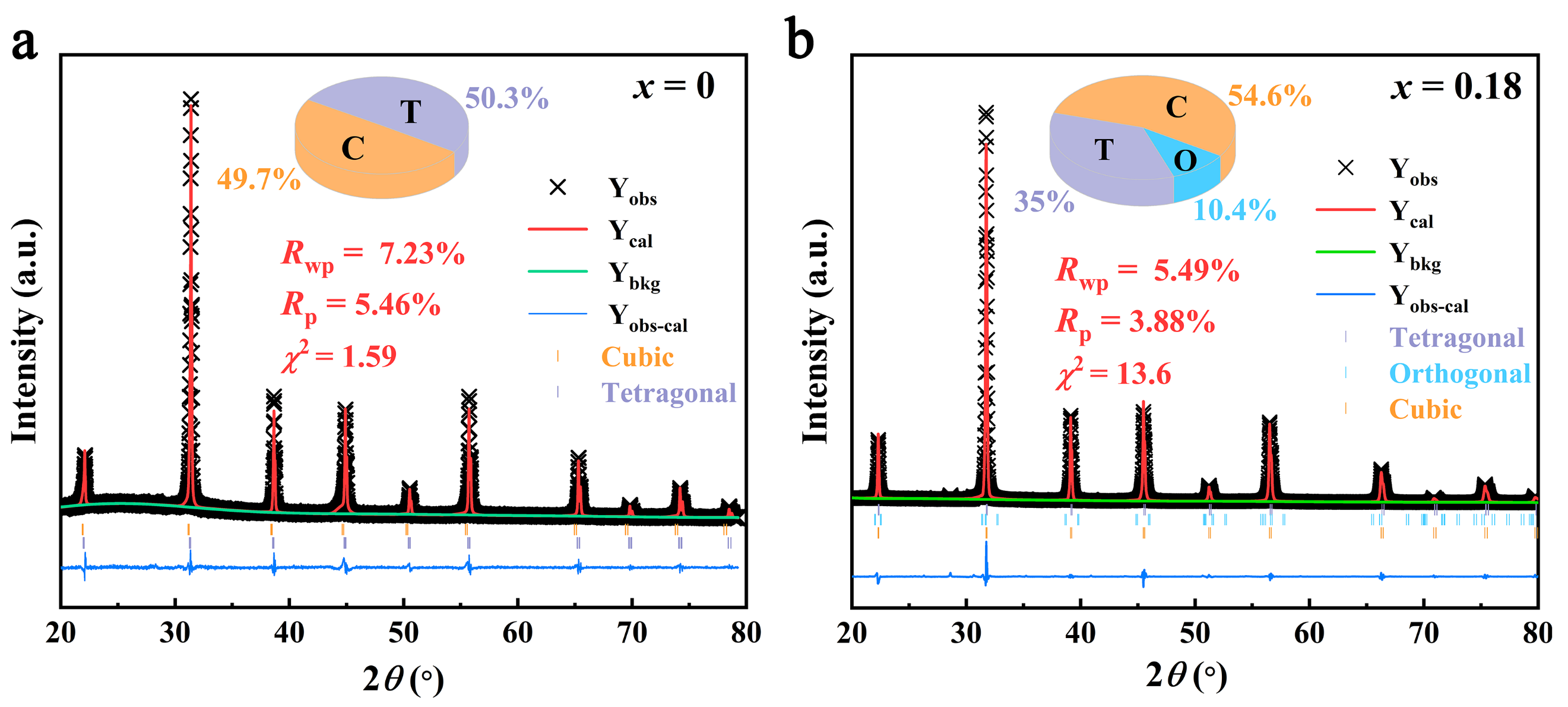


Figure S2. Rietveld refinement of the (a) *x* = 0 ceramic and (b) *x* = 0.18 ceramic.


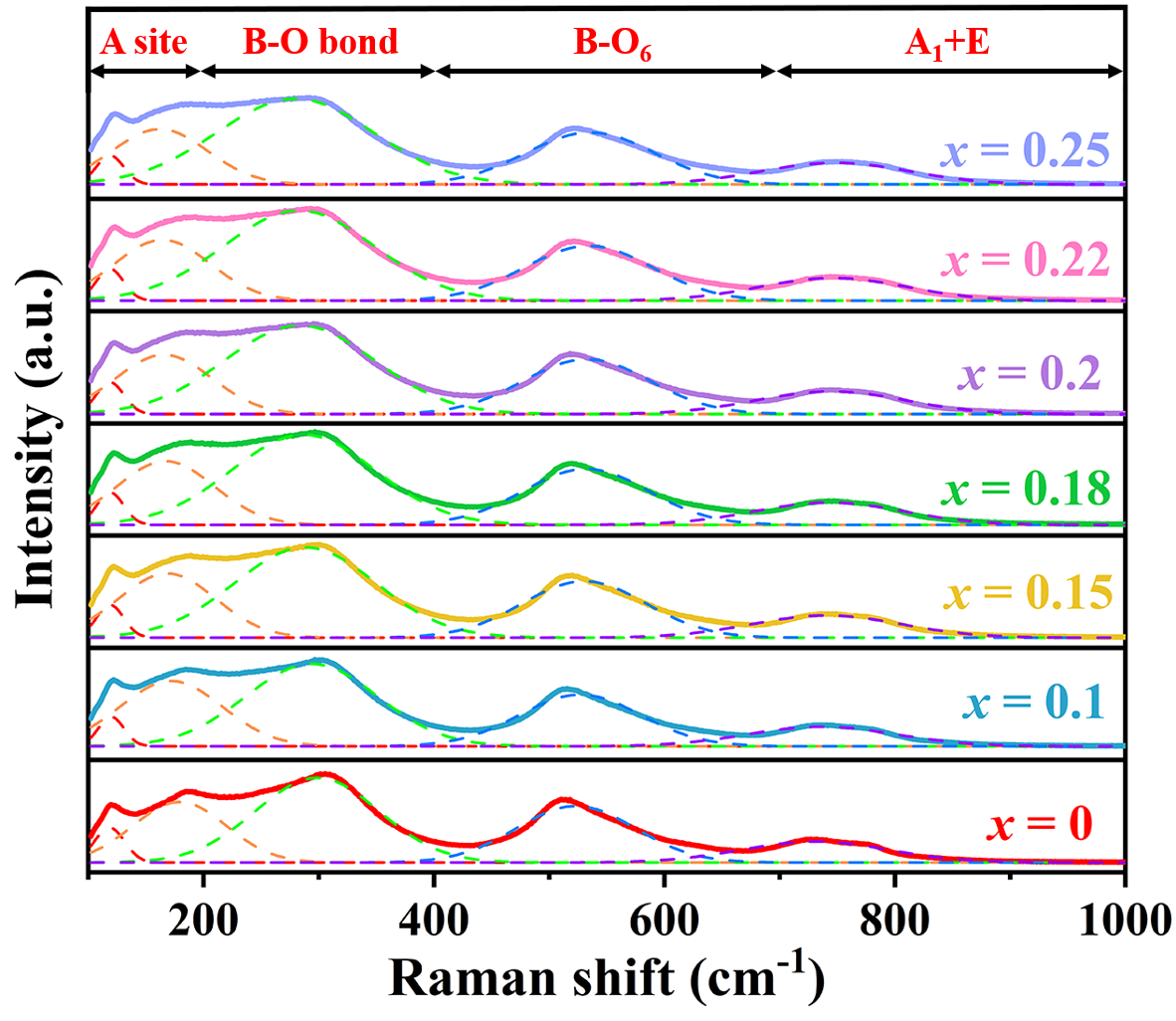


Figure S3. Raman spectra of B_1-_*_x_*C*_x_*T-BMS ceramics.


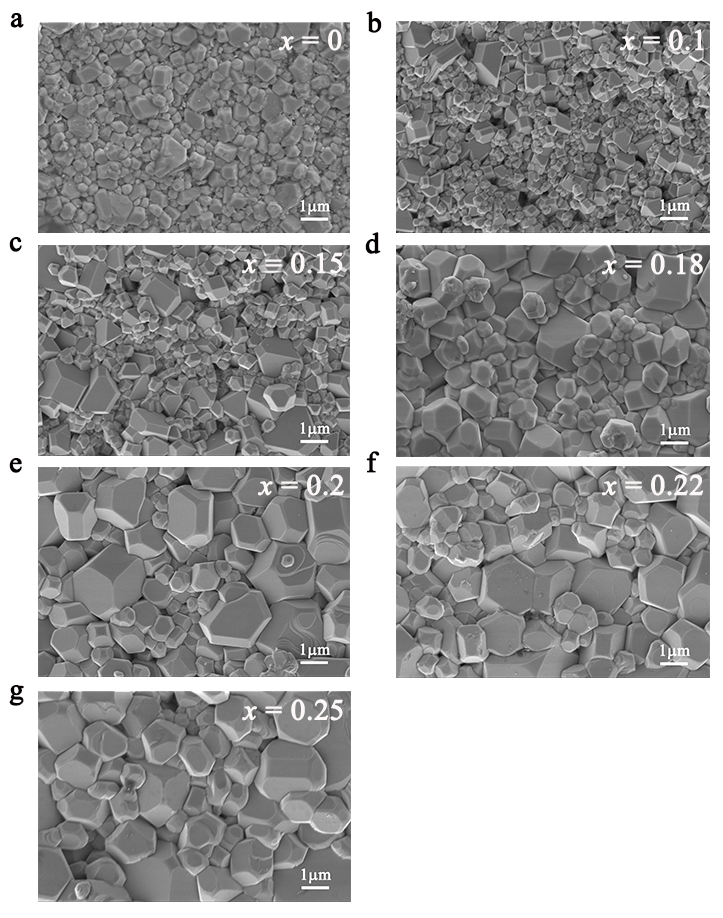


Figure S4. SEM images of B_1-_*_x_*C*_x_*T-BMS ceramics.


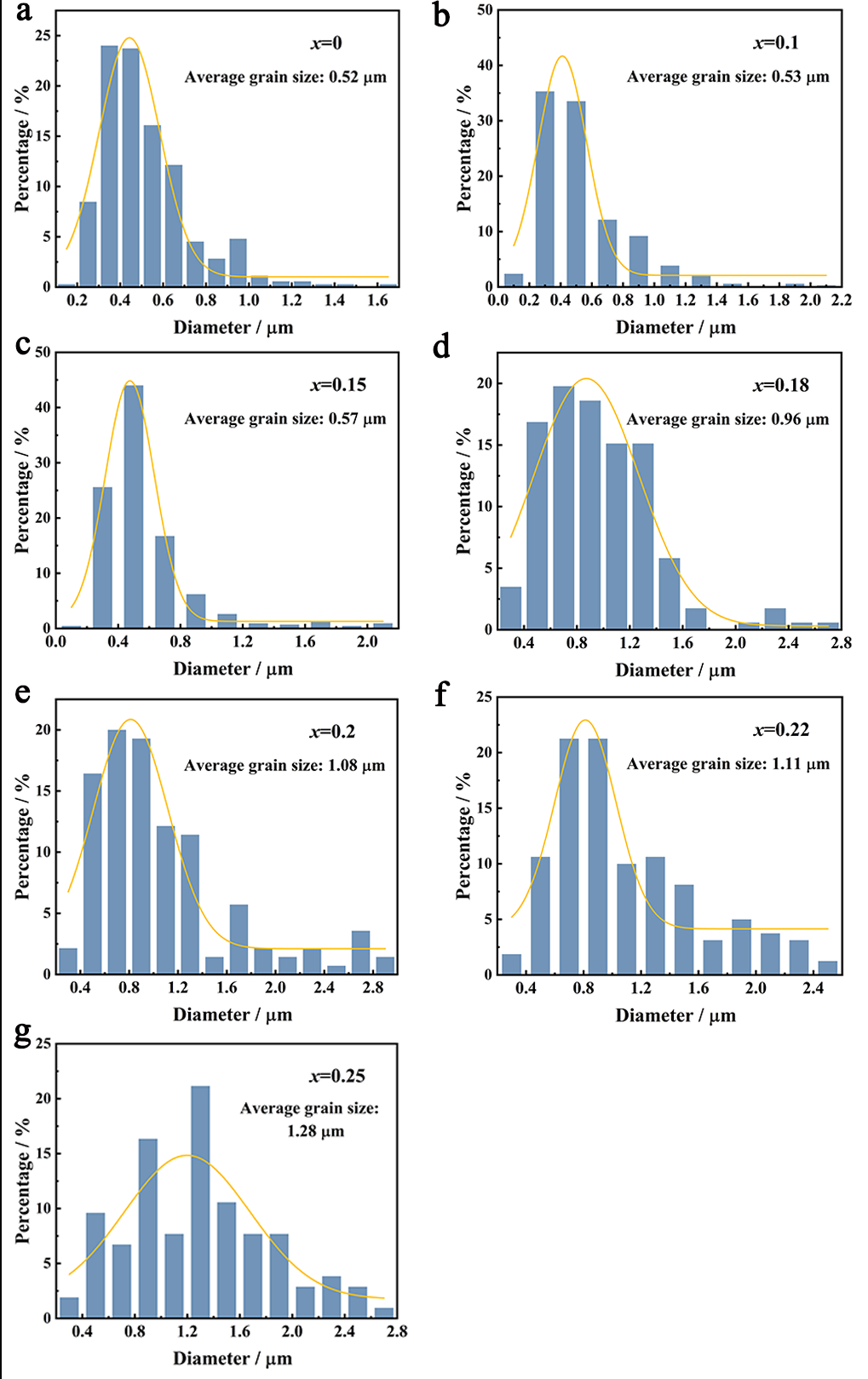


Figure S5. Grain size distribution images of B_1-_*_x_*C*_x_*T-BMS ceramics.


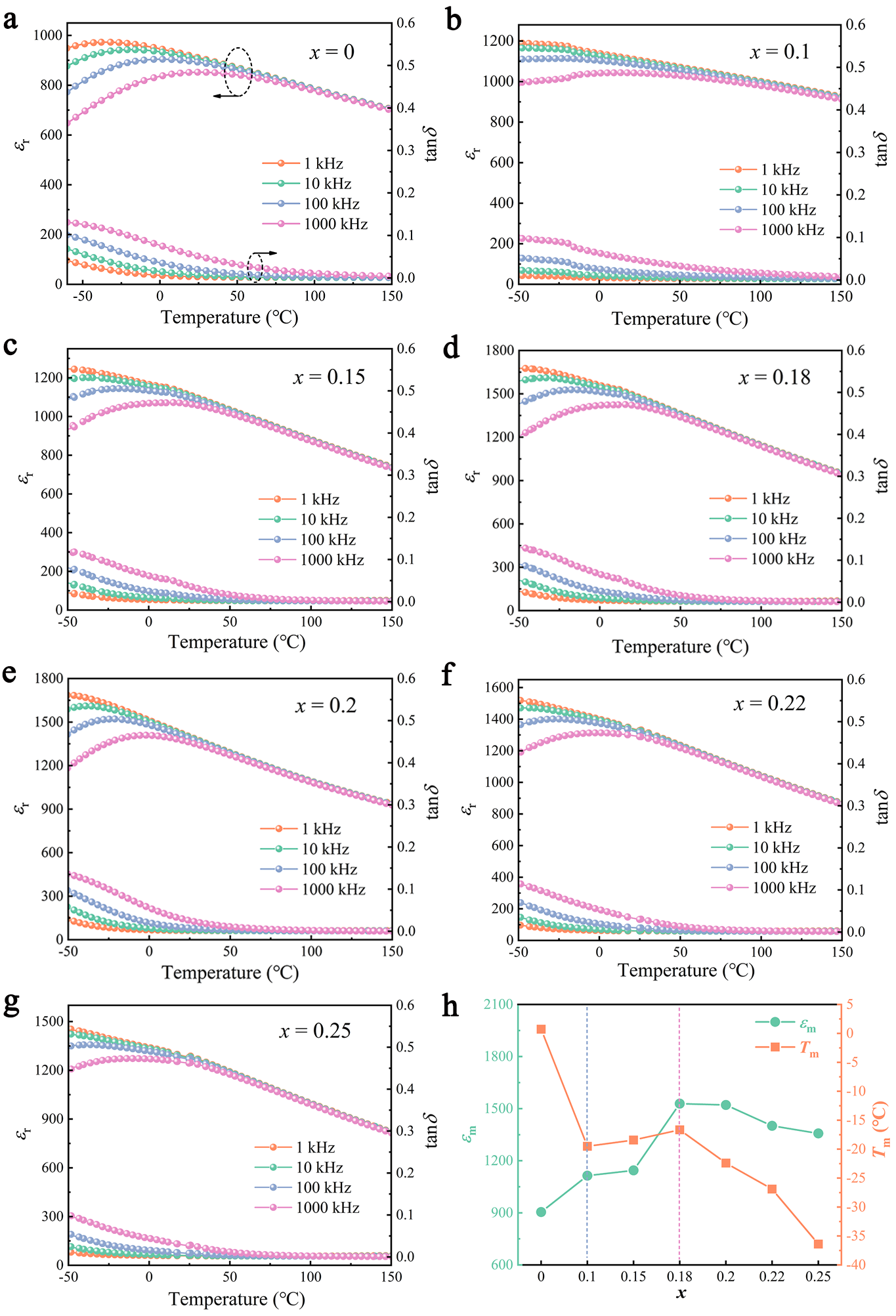


Figure S6. a-g) Dielectric temperature spectrum of B_1-_*_x_*C*_x_*T-BMS ceramics; h) *ε*_m_ and its corresponding *T*_m_ at 100kHz.


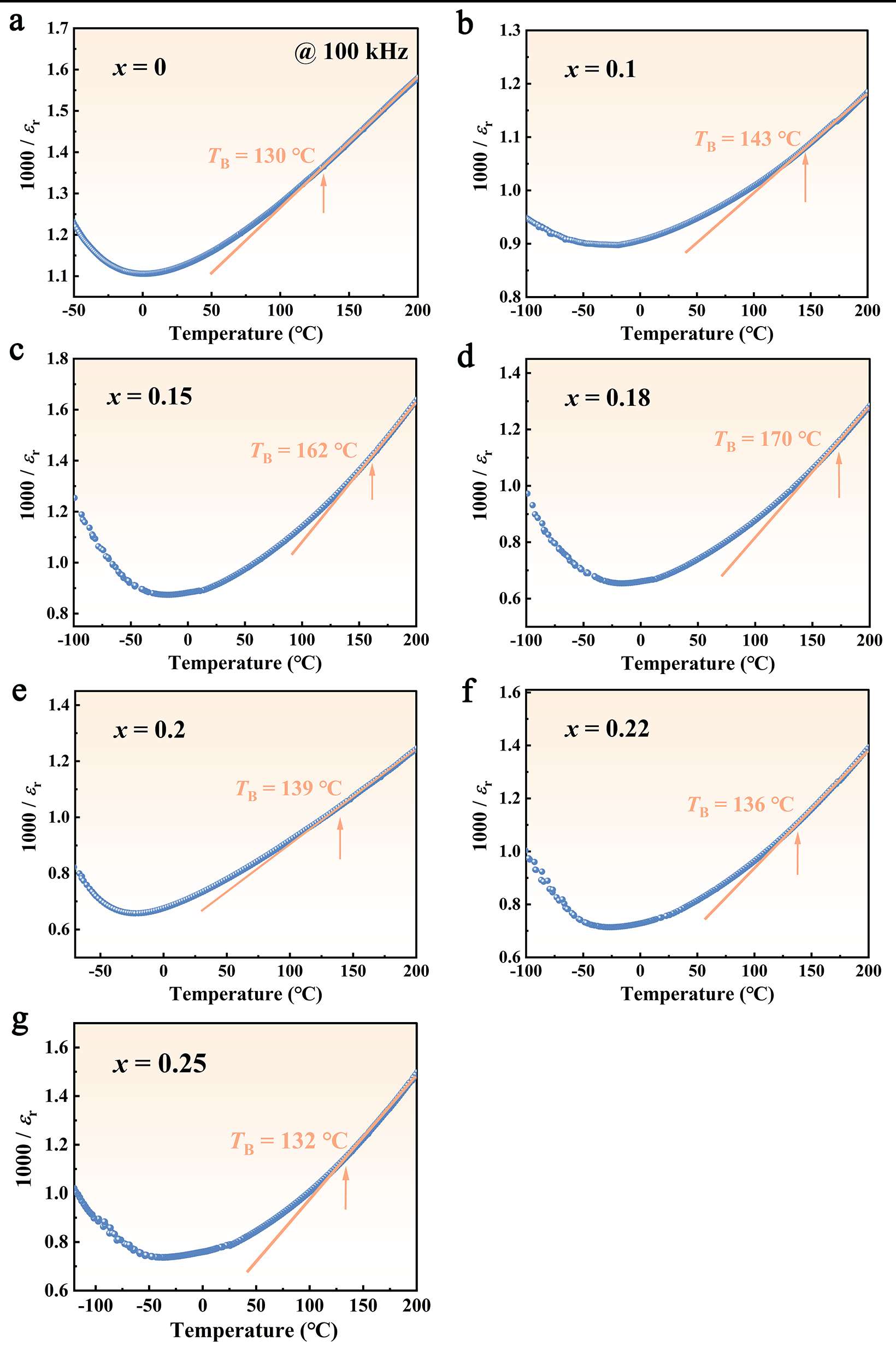


Figure S7. a-g) 1000/*ε*_r_ vs. Temperature curves for all samples.


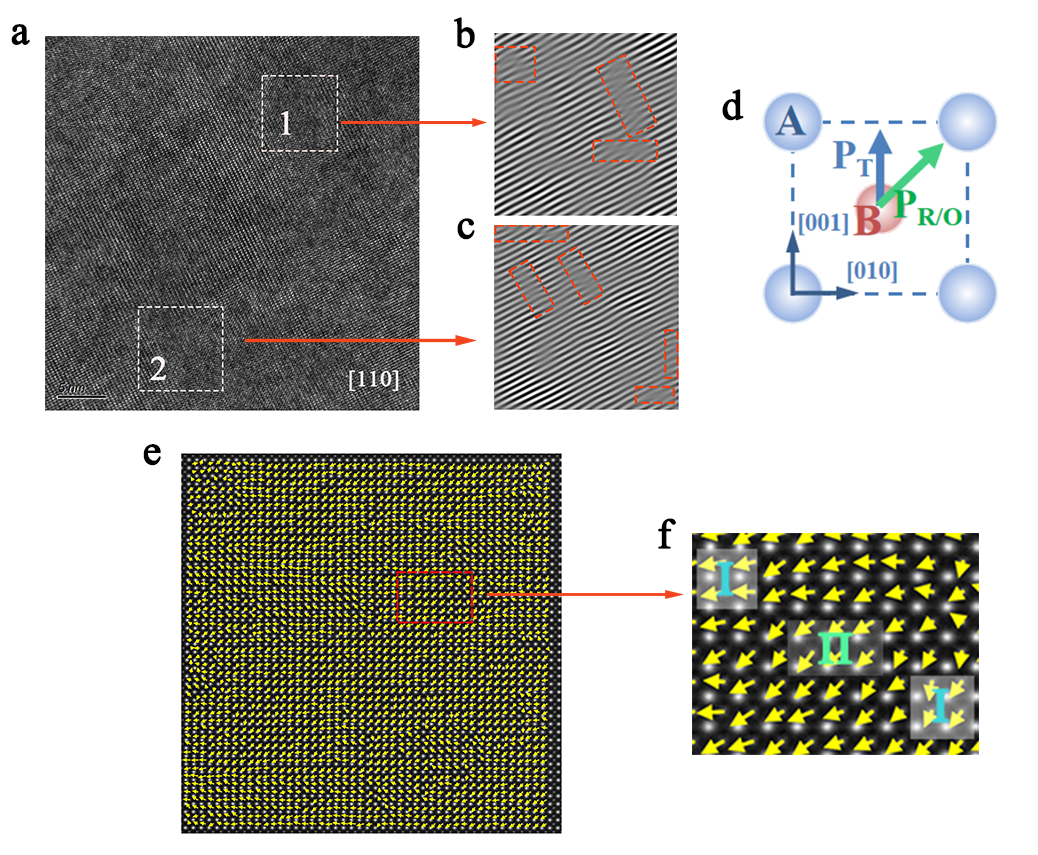


Figure S8. a) High-resolution image of [110]_c_; b, c) the lattice fringe images corresponding to regions 1 and 2 in Figure S4a, with noticeable distortions marked by red dashed boxes; d) enlarged unit cell projection along the [100] direction; e) HAADF STEM polarization vector image along the [110] direction of the *x* = 0.18 sample; f) enlarged view of the marked area (red rectangular area in Figure S4e), showing the transition of polarization vectors from T-phase (Type I) to O-phase (Type Ⅱ) and back to T-phase.


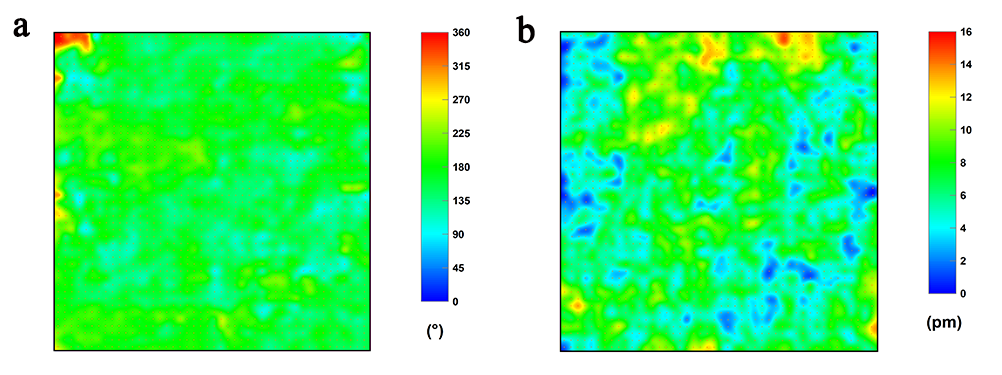


Figure S9. a) Polarization angle mapping of the *x* = 0.18 ceramic along [100]_c_; b) polarization magnitude mapping of *x* = 0.18 ceramic along [100]_c_.


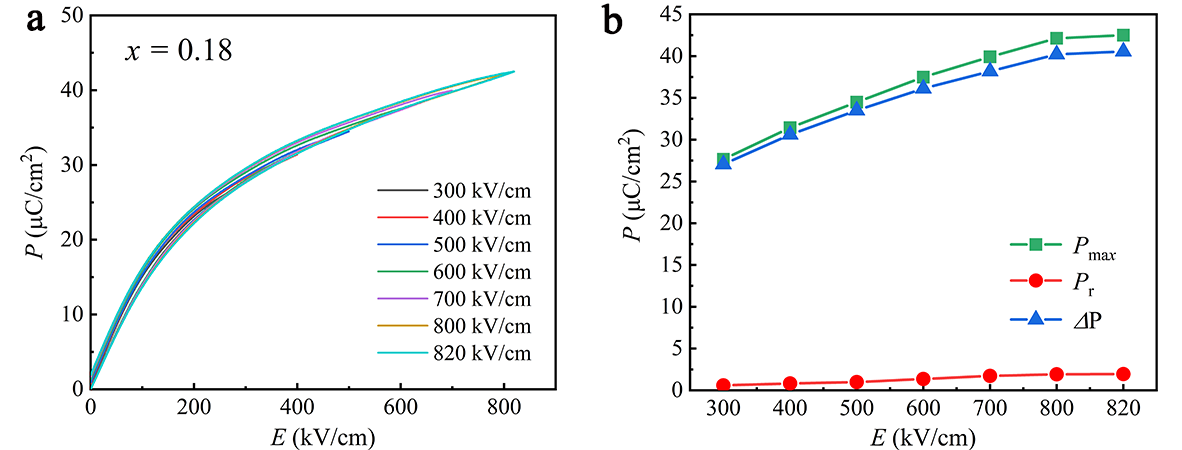


Figure S10. a) Unipolar *P*-*E* loops of the *x* = 0.18 sample with different electric fields; b) the variation of *P*_ma_*_x_*, *P*_r_, and Δ*P*.


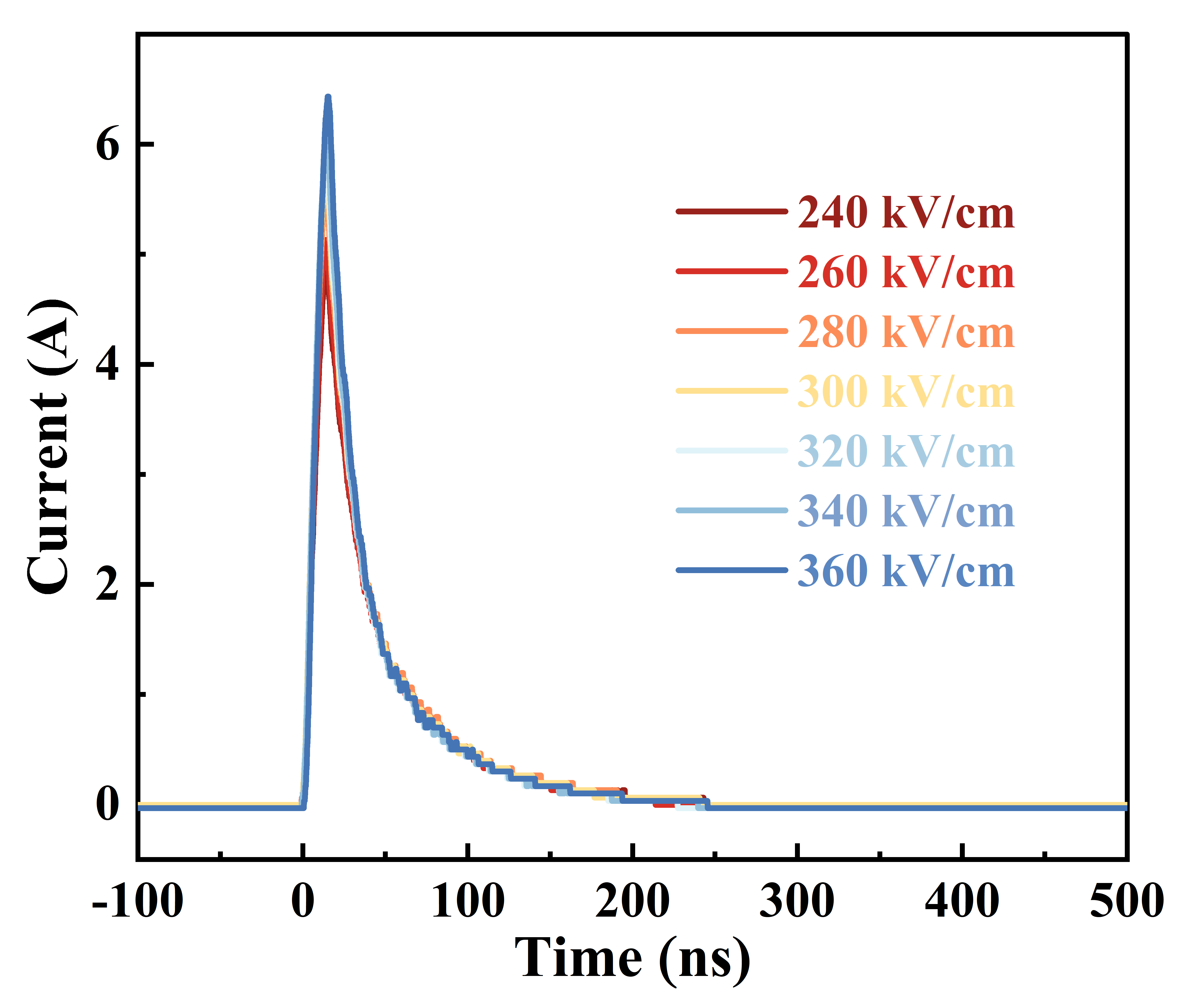


Figure S11. Overdamped current waveform curve for the *x* = 0.18 ceramic.


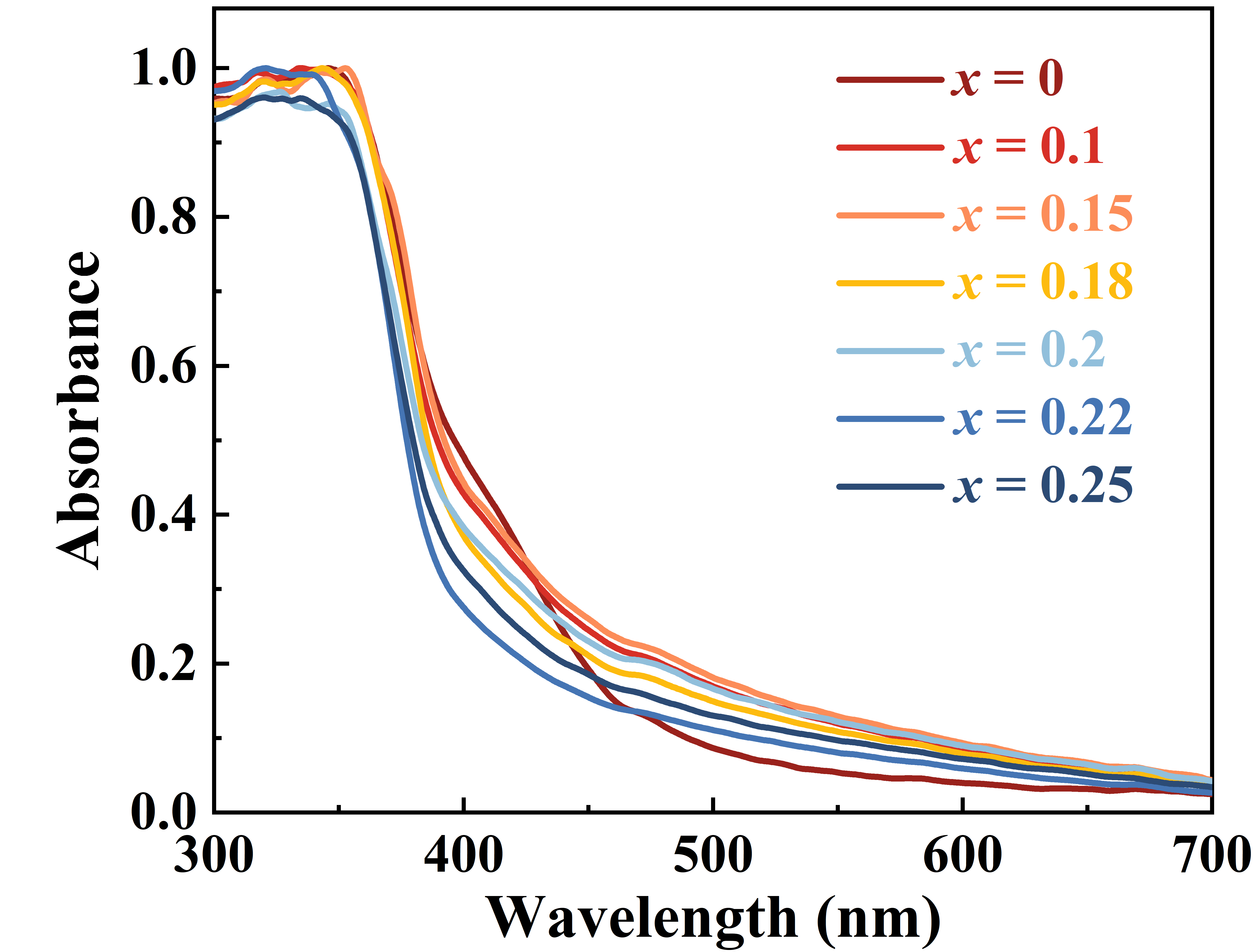


Figure S12. The absorption spectrum curves of all components.


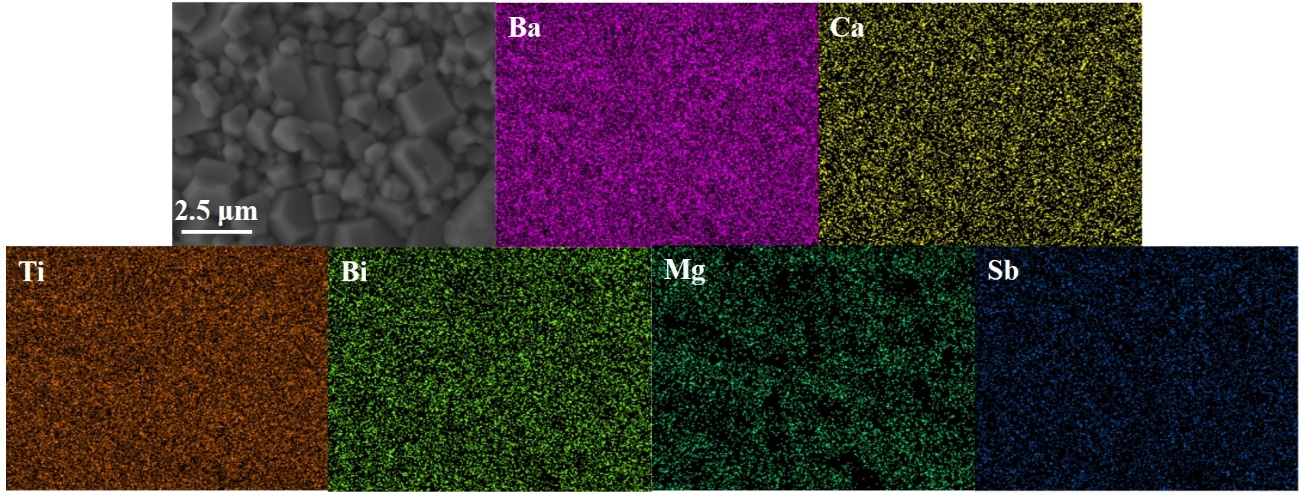


Figure S13. EDS graphics of the *x* = 0.18 ceramic.


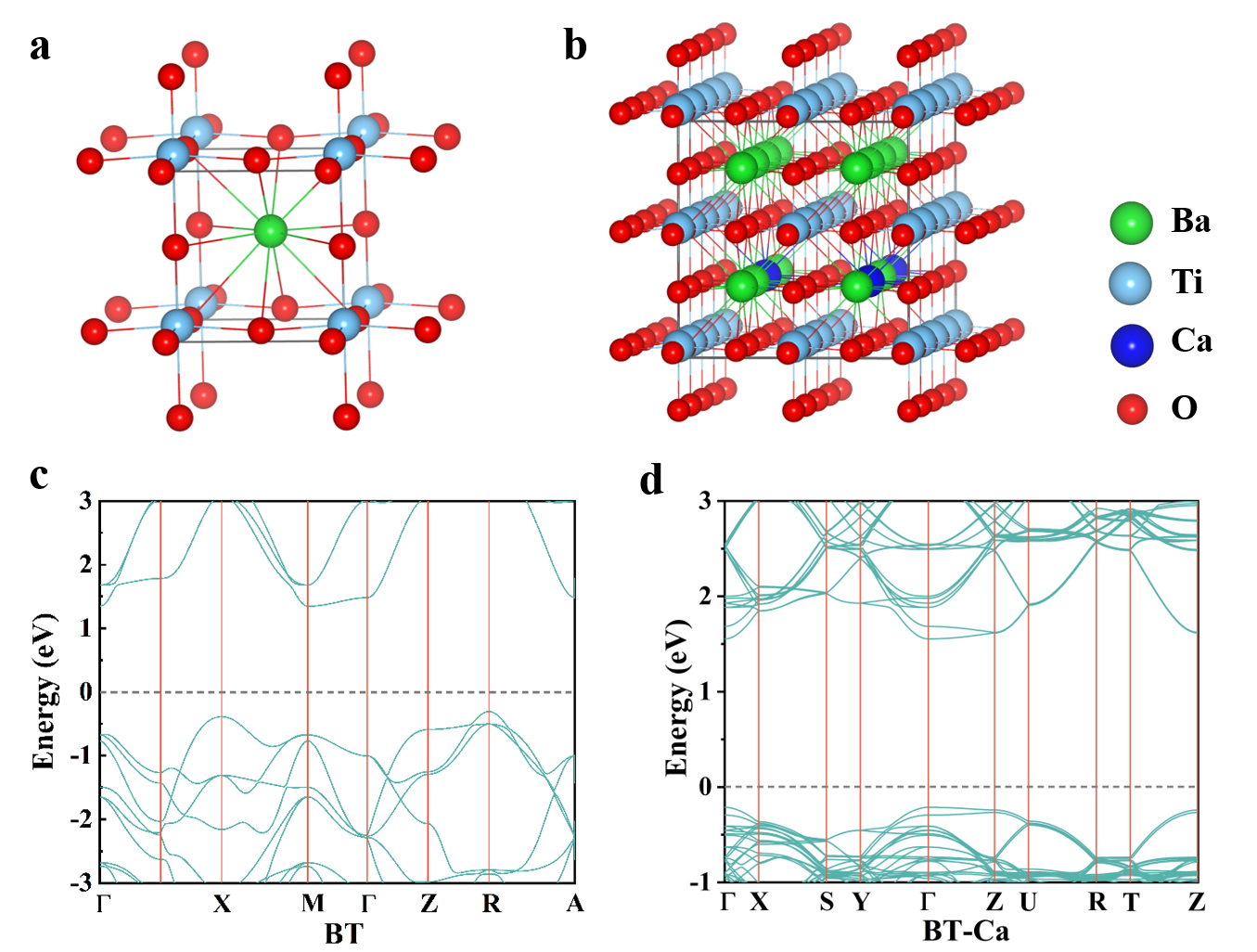


Figure S14. a, b) Structures of (a) BT and (b) BT-Ca (calculated by first principles); c, d) Energy Band diagram of (c) BT and (d) BT-Ca.


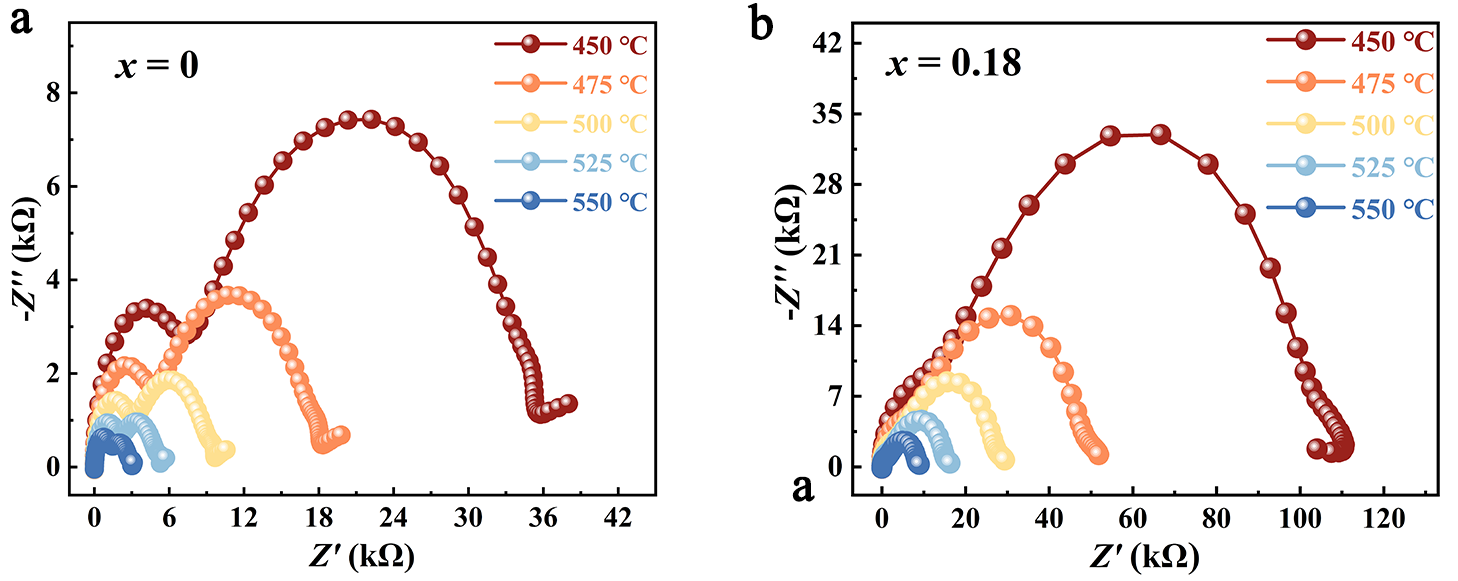


Figure S15. Variable temperature complex impedance mapping of the (a) *x* = 0 ceramic and (b) *x* = 0.18 ceramic.


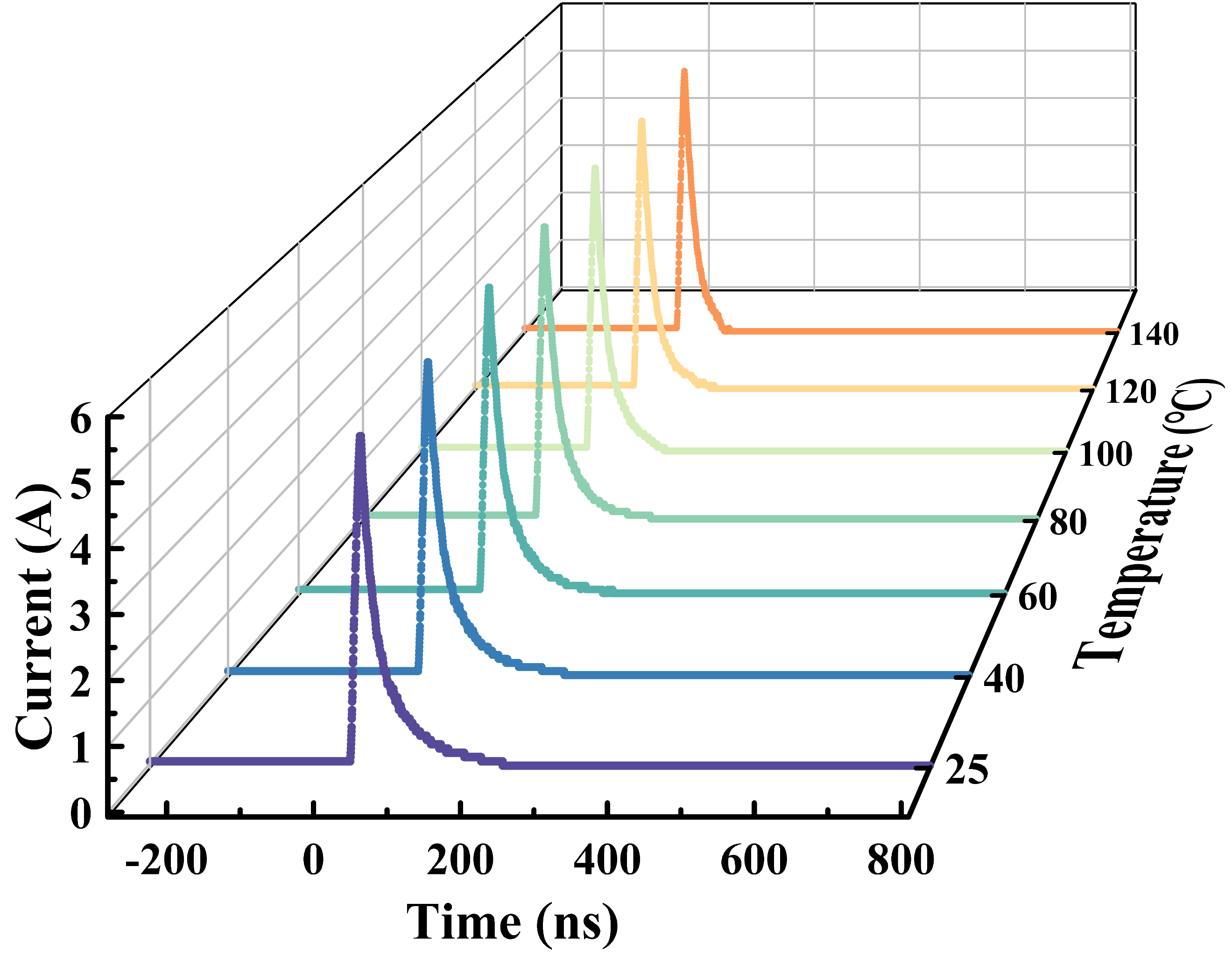


Figure S16. Temperature-related overdamping discharge waveform curve for the *x* = 0.18 ceramic.


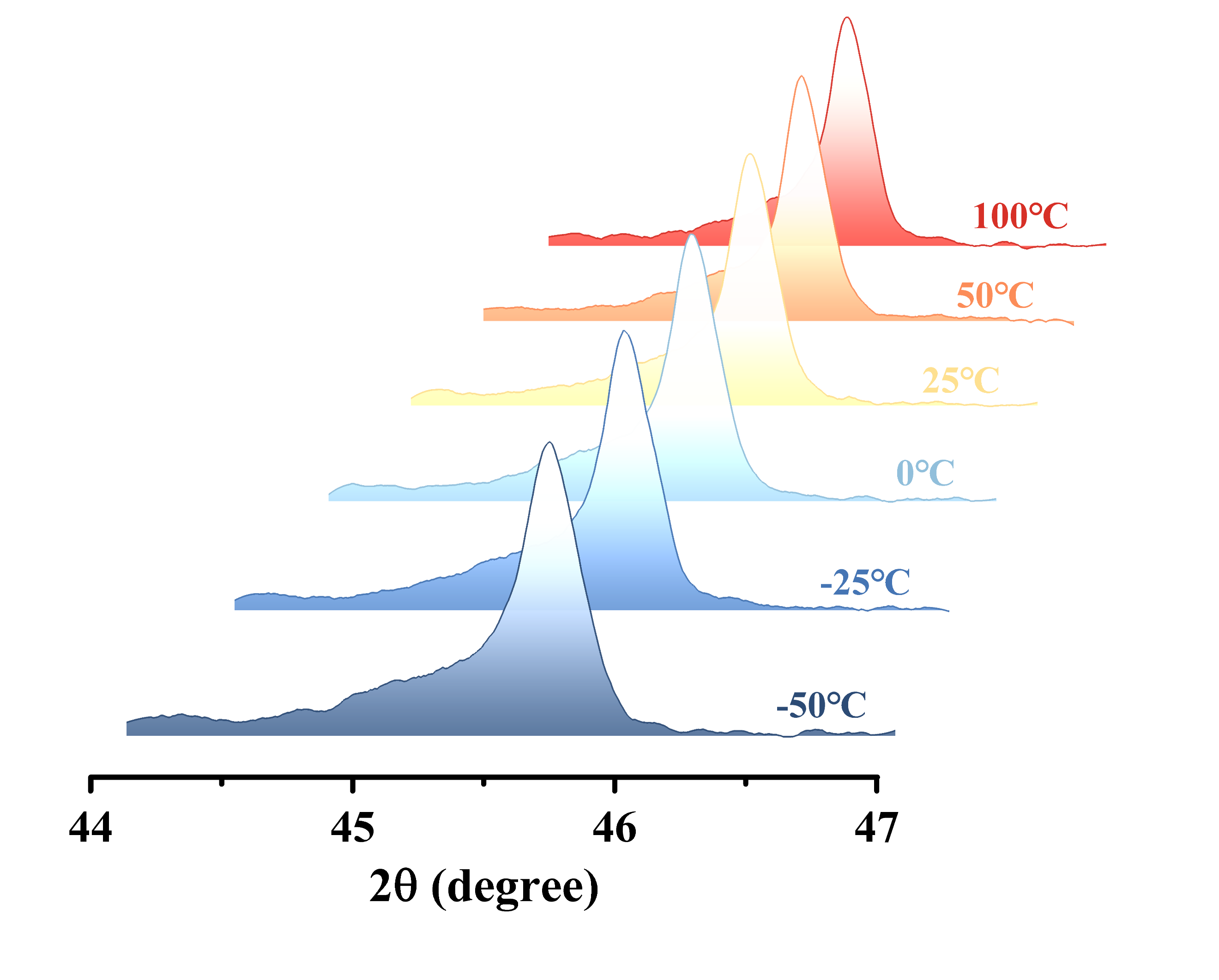


Figure S17. Enlarged image of (200) diffraction peak at different temperatures for the *x* = 0.18 ceramic.

Table S1. Relevant references for Figure 3d, e.^[1-22]^

| **Ceramics** | ***W*_rec_**  **(J cm^-3^)** | ***η* (%)** | ***E*_b_**  **(kV cm^-1^)** | **References** |
| --- | --- | --- | --- | --- |
| BSBiTZ-0.025SLT | 1.92 | 88.32 | 350 | [1] |
| BYH-0.15SZ-0.08BH | 3.46 | 87.34 | 197 | [2] |
| B_0.6_C_0.4_T-BMNT-BNT | 3.59 | 90.86 | 430 | [3] |
| PLSZST-1wt%ZnO | 4.26 | 95.5 | 230 | [4] |
| 0.15BNT | 4.8 | 89 | 525 | [5] |
| 0.825NN-0.1BT-0.075BF | 5 | 82.1 | 300 | [6] |
| BT-0.15BM_5_ | 5.07 | 95.77 | 330 | [7] |
| 0.85BNT-0.11BT-0.04BS | 5.14 | 77.4 | 240 | [8] |
| LLBSCT | 5.16 | 88 | 400 | [9] |
| Yb/Tm-0.03ZTH | 5.39 | 91.97 | 395 | [10] |
| 0.7BT-0.3(BZN-Nb) | 5.96 | 89.5 | 510 | [11] |
| 0.2SNBCT | 6 | 92 | 440 | [12] |
| 0.85BSCT-0.15BS | 6.95 | 86.17 | 500 | [13] |
| 0.4BNT-0.6(Ba_0.15_Sr_0.55_Ca_0.3_)TiO_3_ | 7.34 | 84.4 | 540 | [14] |
| ABN(0.12) | 7.6 | 79 | 522 | [15] |
| BT-BiMZ-0.15BNST | 7.6 | 94 | 595 | [16] |
| 0.85KNN-0.15BNZ | 8.09 | 88.5 | 870 | [17] |
| 0.8BNST-0.2CLT | 8.3 | 80 | 660 | [18] |
| 0.91BST-0.09NBN-VPP | 8.5 | 93.4 | 640 | [19] |
| BSCNT0.30 | 8.9 | 93 | 700 | [20] |
| NBT-BT-0.25CTH | 8.91 | 78.4 | 410 | [21] |
| Gd_0.03_Ba_0.47_Sr_0.455_Sm_0.02_Nb_2_O_6_ | 9 | 84 | 660 | [22] |
| B_0.82_C_0.18_T-BMS | 9.8 | 88.5 | 820 | This work |

**References**

[1] Z. Luo, M. Lin, J. Kong, B. Zhang, W. Li, J. Chen, J. Huang, X. Lei, W. Gong, L. Liu, Achieving Ultrahigh Energy Storage Density for BaTiO_3_-Based Ceramics under Moderate Electric Fields via Regulating Dielectric Permittivity, ACS Applied Materials & Interfaces 2025, 17, 28411-28424. https://doi.org/10.1021/acsami.5c04646

[2] Q. Liao, T. Deng, T. Lu, Z. Liu, N. Narayanan, S. Li, S. Yan, Y. Bao, Y. Liu, G. Wang, Ultrahigh energy storage performance in AN-based superparaelectric ceramics, Chemical Engineering Journal 2024, 488, 150901. https://doi.org/10.1016/j.cej.2024.150901

[3] W. Cao, P. Chen, R. Lin, F. Li, B. Ge, D. Song, Z. Cheng, C. Wang, Boosting energy-storage performance in lead-free ceramics via polyphase engineering in the superparaelectric state, Composites Part B: Engineering 2023, 255, 110630. https://doi.org/10.1016/j.compositesb.2023.110630

[4] M. Zhang, H. Yang, Y. Lin, Q. Yuan, H. Du, Significant increase in comprehensive energy storage performance of potassium sodium niobate-based ceramics via synergistic optimization strategy, Energy Storage Materials 2022, 45, 861-868. https://doi.org/10.1016/j.ensm.2021.12.037

[5] L. Liu, Y. Liu, J. Hao, J. Chen, P. Li, S. Chen, P. Fu, W. Li, J. Zhai, Multi-scale collaborative optimization of SrTiO_3_-based energy storage ceramics with high performance and excellent stability, Nano Energy 2023, 109, 108275. https://doi.org/10.1016/j.nanoen.2023.108275

[6] M. Sun, X. Wang, P. Li, J. Du, P. Fu, J. Hao, W. Li, J. Zhai, Realizing ultrahigh breakdown strength and ultrafast discharge speed in novel barium titanate-based ceramics through multicomponent compounding strategy, Journal of the European Ceramic Society 2023, 43, 974-985. https://doi.org/10.1016/j.jeurceramsoc.2022.11.002

[7] J. Yan, G. Yan, J. Sun, B. Fang, S. Zhang, X. Lu, J. Ding, Improving energy storage properties in (Ba_0.75_Sr_0.1_Bi_0.1_)(Ti_0.9_Zr_0.1_)O_3_ ceramic thick films by adding (Sb_0.5_Li_0.5_)TiO_3_, domain engineering and defect engineering, Journal of Energy Storage 2024, 104, 114549. https://doi.org/10.1016/j.est.2024.114549

[8] X. Zeng, X. Jiang, J. Lin, Q. Lin, S. Wang, Y. Wu, X. Wu, M. Gao, C. Zhao, T. Lin, L. Luo, C. Lin, Excellent low-*E* energy storage and fluorescence temperature sensing features in Bi_0.5_Na_0.5_TiO_3_-based transparent ceramics, Chemical Engineering Journal 2024, 496, 154150. https://doi.org/10.1016/j.cej.2024.154150

[9] H. Zhao, R. Xu, M. Wang, G. Wang, H. Sun, X. Wang, Q. Zhu, X. Wei, Y. Feng, Z. Xu, Excellent Energy Storage Performance of ZnO doped (Pb, La)(Zr, Sn, Ti)O_3_ Based Antiferroelectric Ceramics at an Ultra-Low Sintering Temperature of 940 °C, Advanced Functional Materials 2024, 34, 2316674. https://doi.org/10.1002/adfm.202316674

[10] D. Zeng, P. Nong, M. Xu, Q. Dong, Y. Pan, X. Wang, J. Wang, S. Yang, H. Zhou, X. Chen, Relaxor ferroelectric ceramics with excellent energy storage density obtained from BT-based ceramics, Journal of Power Sources 2023, 580, 233454. https://doi.org/10.1016/j.jpowsour.2023.233454

[11] A. Xie, T. Hu, J. Lei, Y. Zhang, X. Wei, Z. Fu, R. Zuo, Local Isomeric Polar Nanoclusters Enabled Superior Capacitive Energy Storage Under Moderate Fields in NaNbO_3_-Based Lead-Free Ceramics, Small 2024, 20, 2309796. https://doi.org/10.1002/smll.202309796

[12] K. Chen, R. Zhao, L. Zhang, X. Zhu, W. Li, X. Tang, Z. Jian, Y. Liang, Y. Zhao, Y. Jiang, X. Guo, K. Yan, Achieving near-zero energy loss in 0.85BaTiO_3_-0.15Bi(Mg_0.2_Hf_0.2_Ni_0.2_Zn_0.2_Ta_0.2_)O_3_ ceramic through high-entropy and superparaelectric engineering, Ceramics International 2025, 51, 29487-29498. https://doi.org/10.1016/j.ceramint.2025.04.153

[13] Y. Xu, X. He, S. Gao, T. Li, D. Pang, Enhanced energy storage performance of BiScO_3_ modified Bi_0.5_Na_0.5_TiO_3_-BaTiO_3_ lead-free ferroelectric ceramics, Journal of Alloys and Compounds 2024, 1008, 176548. https://doi.org/10.1016/j.jallcom.2024.176548

[14] P. Gao, R. Zhang, C. Liu, H. Wang, W. Geng, J. Zhang, Z. Sun, Y. Tian, X. Ren, Significant enhancement of comprehensive energy storage performance in BaTiO_3_-based ceramics through high-entropy design, Journal of the European Ceramic Society 2025, 45, 117401. https://doi.org/10.1016/j.jeurceramsoc.2025.117401

[15] C. Long, W. Zhou, L. Liu, H. Song, H. Wu, K. Zheng, W. Ren, X. Ding, Achieving excellent energy storage performances and eminent charging-discharging capability in donor (1-*x*)BT-*x*(BZN-Nb) relaxor ferroelectric ceramics, Chemical Engineering Journal 2023, 459, 141490. https://doi.org/10.1016/j.cej.2023.141490

[16] X. Nie, R. Jing, Y. Yang, F. Chen, M. Meng, Y. Yan, D. Alikin, V. Shur, Y. Yan, L. Zhang, L. Jin, Synergistic enhancement of energy storage performance in BNT-based ceramics through the co-doping of multiple A-site ions, Chemical Engineering Journal 2025, 507, 160500. https://doi.org/10.1016/j.cej.2025.160500

[17] S. Yang, D. Zeng, Q. Dong, Y. Pan, X. Chen, X. Li, H. Zhou, Lead-free BaTiO_3_-based composite ceramics with ultra-high energy storage performance via synergistic modulation of polarization and breakdown strength, Journal of Power Sources 2025, 632, 236399. https://doi.org/10.1016/j.jpowsour.2025.236399

[18] L. Tang, Z. Yu, Z. Pan, J. Zhao, Z. Fu, X. Chen, H. Li, P. Li, J. Liu, J. Zhai, Giant Energy Storage Density with Antiferroelectric-Like Properties in BNT-Based Ceramics via Phase Structure Engineering, Small 2023, 19, 2302346. https://doi.org/10.1002/smll.202302346

[19] W. Wang, Y. Yang, J. Qian, W. Shi, Y. Huang, R. Jing, L. Zhang, Z. Pan, V. Laletin, V. Shur, J. Zhai, L. Jin, Advancing energy storage properties in barium titanate-based relaxor ferroelectric ceramics through a stagewise optimization strategy, Chemical Engineering Journal 2024, 488, 151043. https://doi.org/10.1016/j.cej.2024.151043

[20] J. Wu, H. Tan, H. Qi, H. Yu, L. Chen, W. Li, J. Chen, High Energy Storage Performance in BiFeO_3_-Based Lead-Free High-Entropy Ferroelectrics, Small 2024, 20, 2400997. https://doi.org/10.1002/smll.202400997

[21] Y. Gao, W. Qiao, X. Lou, Z. Song, X. Zhu, L. He, B. Yang, Y. Hu, J. Shao, D. Wang, Z. Chen, S. Zhang, Ultrahigh Energy Storage in Tungsten Bronze Dielectric Ceramics Through a Weakly Coupled Relaxor Design, Advanced Materials 2024, 36, 2310559. https://doi.org/10.1002/adma.202310559

[22] X. Zeng, J. Lin, Y. Chen, S. Wang, P. Zhou, F. Yu, X. Wu, M. Gao, C. Zhao, T. Lin, L. Luo, C. Lin, Superior Energy Storage Capability and Fluorescence Negative Thermal Expansion of NaNbO_3_-Based Transparent Ceramics by Synergistic Optimization, Small 2024, 20, 2309992. https://doi.org/10.1002/smll.202309992
